# Structure of the TELO2-TTI1-TTI2 complex and its function in TOR recruitment to the R2TP chaperone

**DOI:** 10.1016/j.celrep.2021.109317

**Published:** 2021-07-06

**Authors:** Mohinder Pal, Hugo Muñoz-Hernandez, Dennis Bjorklund, Lihong Zhou, Gianluca Degliesposti, J. Mark Skehel, Emma L. Hesketh, Rebecca F. Thompson, Laurence H. Pearl, Oscar Llorca, Chrisostomos Prodromou

**Affiliations:** 1 Genome Damage and Stability Centre, School of Life Sciences, University of Sussex, Falmer, Brighton BN1 9RQ, UK; 2 Spanish National Cancer Research Centre (CNIO), Melchor Fernández Almagro 3, 28029 Madrid, Spain; 3 MRC Laboratory of Molecular Biology, Francis Crick Avenue, Cambridge Biomedical Campus, Cambridge CB2 0QH, UK; 4 Astbury Centre for Structural Molecular Biology, School of Molecular and Cellular Biology, Faculty of Biological Sciences, University of Leeds, Leeds LS2 9JT, UK; 5 Division of Structural Biology, Institute of Cancer Research, Chester Beatty Laboratories, 237 Fulham Road, London SW1E 6BT, UK

**Keywords:** R2TP, TTT, TELO2, TTI1, TTI2, RUVBL1, RUVBL2, HSP90 chaperone, mTOR, PIKK

## Abstract

The R2TP (RUVBL1-RUVBL2-RPAP3-PIH1D1) complex, in collaboration with heat shock protein 90 (HSP90), functions as a chaperone for the assembly and stability of protein complexes, including RNA polymerases, small nuclear ribonucleoprotein particles (snRNPs), and phosphatidylinositol 3-kinase (PI3K)-like kinases (PIKKs) such as TOR and SMG1. PIKK stabilization depends on an additional complex of TELO2, TTI1, and TTI2 (TTT), whose structure and function are poorly understood. The cryoelectron microscopy (cryo-EM) structure of the human R2TP-TTT complex, together with biochemical experiments, reveals the mechanism of TOR recruitment to the R2TP-TTT chaperone. The HEAT-repeat TTT complex binds the kinase domain of TOR, without blocking its activity, and delivers TOR to the R2TP chaperone. In addition, TTT regulates the R2TP chaperone by inhibiting RUVBL1-RUVBL2 ATPase activity and by modulating the conformation and interactions of the PIH1D1 and RPAP3 components of R2TP. Taken together, our results show how TTT couples the recruitment of TOR to R2TP with the regulation of this chaperone system.

## Highlights

• TELO2-TTI1-TTI2 (TTT) forms a direct complex with RUVBL1-RUVBL2 (R2)
• TTT interacts with two consecutive DII domains in the heterohexameric R2 ring
• TTT inhibits R2 ATPase and antagonizes RPAP3/Tah1p–PIH1D1/Pih1p engagement
• TTI1-TTI2 binds the kinase region of mTOR but does not inhibit its catalytic activity

## Graphical abstract

**Figure:**
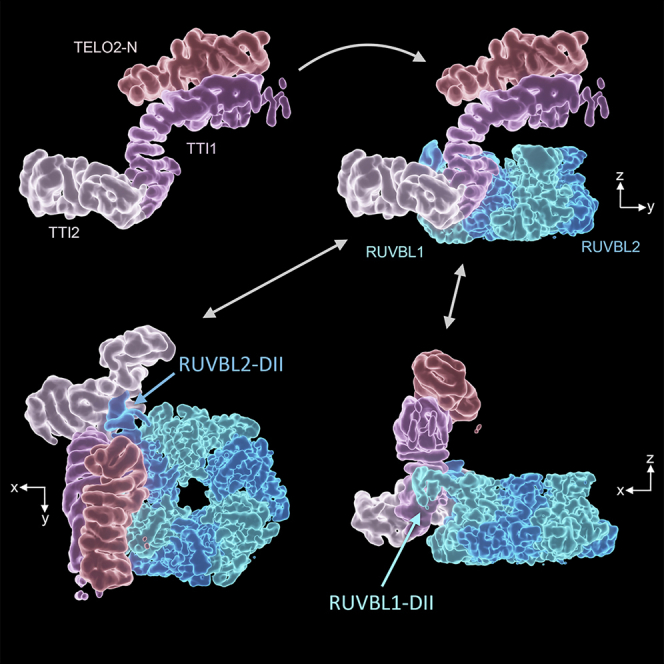

## Teaser

Pal et al. report the cryo-EM structure of the TELO2-TTI1-TTI2 (TTT) complex bound to a heterohexameric ring of the RUVBL1-RUVBL2 (R2) AAA+ ATPases, showing a direct interaction of the HEAT repeats of TTI1 and TTI2 with R2 DII domains. TTT binding inhibits R2 ATPase activity and facilitates mTOR recruitment.

## Introduction

The R2TP complex is a specialized co-chaperone of heat shock protein 90 (HSP90), consisting of an alternating heterohexameric ring of two AAA+ ATPases, RUVBL1 (also known as Pontin, TIP49, and TIP49a, and in yeast as Rvb1p) and RUVBL2 (also known as Reptin, TIP48, and TIP49b, and in yeast as Rvb2p), a TPR-domain protein, RPAP3 (Tah1p in yeast), and a PIH-domain protein, PIH1D1 (Pih1p in yeast) (Martino et al., 2018; Rivera-Calzada et al., 2017). R2TP has been implicated in many biological processes, including the assembly, activation, and stabilization of phosphatidylinositol 3 kinase (PI3K)-related kinases (PIKKs) such as mTORC1, SMG1, ATM, DNA-PK, and ATR/ATRIP, the assembly of RNA polymerase II and small nucleolar ribonucleoprotein particles (snoRNPs), and in the assembly of axonemal dyneins (von Morgen et al., 2015). Most recently, R2TP has been implicated in the function and assembly of replisome and nucleocapsids of RNA viruses responsible for measles and Ebola (Katoh et al., 2019; Morwitzer et al., 2019)

The structures of yeast and human R2TP complexes have been analyzed by cryoelectron microscopy (cryo-EM) (Martino et al., 2018; Rivera-Calzada et al., 2017; Tian et al., 2017). RUVBL1/Rvb1p and RUV/Rvb2p (henceforth R2) form a heterohexamer through interaction of the alternating AAA domains of each subunit. Domain II (DII) from each subunit projects from the hexameric ring and defines the DII face of R2, while the AAA-ATPase domains define the opposing AAA face. In all R2 complexes studied so far, other associated proteins predominantly bind to the DII face of the ring, and in yeast and human R2TP complexes, the HSP90-recruiting TPR domains of RPAP3/Tah1p and the client-recruiting PIH-domain-containing RPAP3/Tah1p-PIH1D1/Pih1p complex (henceforth TP), are located on this face of the ring. In human R2TP, RPAP3, a much larger protein than the yeast homolog Tah1p, contains a unique C-terminal α-helical domain that binds to the AAA side of each RUVBL2 subunit. This RUVBL2-binding domain (RBD) provides a tight anchor for RPAP3 in complex with PIH1D1, while allowing for considerable flexibility of the TPR and N-terminal domains of RPAP3 at the DII face of R2. Since each R2TP complex contains three copies of RUVBL2, up to three RPAP3-PIH1D1 complexes can bind per R2TP, but although flexibly tethered, only one PIH1D1 at a time can interact directly to R2. One molecule of PIH1D1 binds to the DII domain of one RUVBL2 subunit, inducing conformational changes that facilitate nucleotide exchange in this subunit and promote ATP turnover (Munoz-Hernandez et al., 2019).

Recruitment of PIKK proteins to the R2TP complex requires an additional adaptor complex formed by TELO2 (Takai et al., 2007) and its associated proteins TTI1 and TTI2 (Takai et al., 2010), known as TTT. Recruitment of TTT to R2TP is thought to be mediated through interaction of a CK2-phosphorylated acidic motif in the flexible linker connecting the N- and C-terminal domains of TELO2, with the PIH domain of PIH1D1 (Hořejší et al., 2014; Horejsí et al., 2010; Pal et al., 2014). TELO2 interaction with PIKKs has itself been suggested to be dependent on HSP90, although the mechanistic basis for this has not been defined (Takai et al., 2010).

We show that contrary to expectations TTT can be directly recruited to R2, and we have determined the structure of the human R2-TTT complex by single-particle cryo-EM. The structure permits atomic modeling of the RUVBL1 and RUVBL2 and tracing of the α-helical repeats that form the solenoid structures of the TTT proteins. Our data reveal a direct engagement of the RUVBL1-RUVBL2 heterohexamer by the TTT complex that is independent of the RPAP3-PIH1D1 (TP) components. TTT interacts with two DII domains from consecutive RUVBL1 and RUVBL2 subunits, and this constrains the conformational flexibility of the DII domains of the RUVBL proteins in the ADP-bound state, inhibits the conformationally coupled activity of the RUVBL AAA+ ATPase, and disturbs the engagement of PIH1D1 to the DII domains. We show that TTT is fully competent to bind a strongly R2TP-dependent PIKK client, TOR, but does so without inhibiting TOR kinase activity. Taken together, our cryo-EM structures and biochemical experiments reveal that TTT couples the recruitment of TOR to the R2TP chaperone with the regulation of the chaperone.

## Results

### Cryo-EM of the RUVBL1-RUVBL2-TTT complex

**Figure 1:**
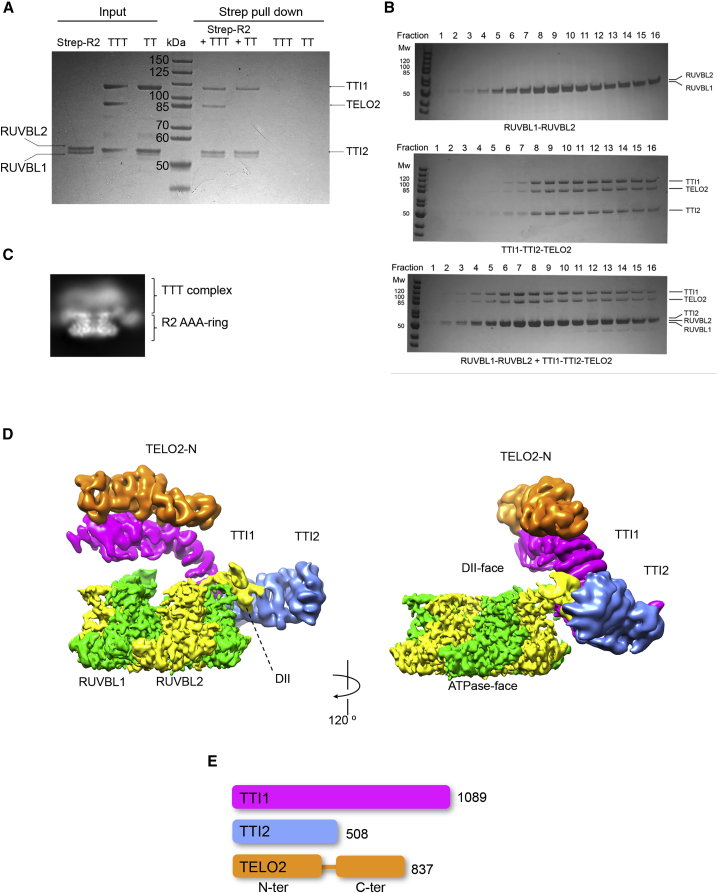
Cryo-EM of the human RUVBL1-RUVBL2-TTT complex (A) Coomassie-stained SDS-PAGE gel showing analysis of interaction of RUVBL1-RUVBL2 (R2), TTI1-TTI2-TELO2 (TTT), and TTI1-TTI2 (TT) subcomplexes. Pull-down on the strep-tag attached to the C terminus of RUVBL2 co-precipitates TTT and TT subcomplexes, showing that neither RPAP3, PIH1D1, nor TELO2 is required to couple TTI1-TTI2 to R2. TTT and TT are not pulled down in the absence of R2. (B) Coomassie-stained SDS-PAGE of fractions from a size-exclusion chromatography column for the human RUVBL1-RUVBL2 complex, R2, (top panel), the TTT complex (middle panel), and the mixture of R2 and TTT (bottom panel). In the mixture, both R2 and TTT elute in earlier fractions than when loaded separately. (C) Representative 2D average of R2-TTT cryo-EM data. (D) Views of human R2-TTT volume. RUVBL1-RUVBL2 hexamer, green and yellow respectively. Three distinct α-helical segments (TTI1, magenta; TTI2, blue; and TELO2-N, orange) are identifiable in the cryo-EM reconstruction after multi-body refinement of the R2-TTT complex. (E) Schematic showing the relative lengths of the TTT components TTI1, TTI2, and TELO2. All three proteins are known or thought to be entirely composed of HEAT repeats, which are interrupted by an ~70-residue flexible linker in TELO2 that connects the N- and C-terminal domains.

Although current models suggest that TTT recruitment to R2TP is mediated by the specific interaction of PIH1D1 with a CK2 phosphorylation site on TELO2 (Hořejší et al., 2014), we found that we could readily pull down complexes of TELO2-TTI1-TTI2 with strep-tagged RUVBL1-RUVBL2 (R2-TTT) in the absence of RPAP3 and PIH1D1 (Figure 1A) and that R2 and TTT co-migrated in size-exclusion chromatography as a stable complex (Figure 1B). The TTI1-TTI2 subcomplex (henceforth TT) was also co-precipitated by RUVBL1-RUVBL2 (R2-TT), demonstrating that TELO2 was also not required for complex formation (Figure 1A).

The assembled R2-TTT complex was vitrified by plunging into liquid ethane, and Titan Krios microscopes equipped with Gatan K2 detectors were used to collect cryo-EM data. 2D averages showed that TTT occupies the DII face of the RUVBL1-RUVBL2 ring (Figure 1C). First observations during 3D refinement of the cryo-EM data revealed that TTI1-TTI2-TELO2 binds to the DII domains of consecutive RUVBL1 and RUVBL2 subunits, and that we could saturate each R2 ring with up to three TTTs by increasing the TTT-versus-R2 ratio during incubation. We exploited the presence of several copies of TTT per particle making use of a symmetry expansion strategy (see STAR Methods and Figure S1). We also observed that TTT attaches flexibly to the DII domains and we split the volume into two bodies. One body contained the ATPase ring of R2 without the flexible DII domains, and this refined to a resolution of 3.4 Å. The second body included TTT and the two DII domains making direct contact with TTT, and this reached an average resolution of 5.0 Å (Figure S2). Then we built a composite map combining both bodies (Figure 1D).

In the cryo-EM map we could readily identify the heterohexameric ring formed by RUVBL1 and RUVBL2, but, in addition, there is a strong body of density at the edge of the ring, projecting over the face that bears the RUVBL-DII domains, corresponding to TTT (Figure 1D). TELO2, TTI1, and TTI2 are known or predicted to contain substantial regions of HEAT repeat (Kaizuka et al., 2010; Takai et al., 2010), and the density for TTT appears as a large curved arc containing more than 30 α helices in a solenoid arrangement that becomes increasingly poorly defined at one end (magenta), a smaller segment of ∼20 α helices that lies on top of the large segment toward the less well-ordered end (orange), and a second shorter curved arc in which at least 15 helices can be identified, which abuts the well-ordered end of the large arc (blue) (Figure 1D).

Based on its size (Figure 1E), we identified the larger segment as TTI1; however, the attribution of the two smaller segments as TTI2 or TELO2 was less clear. We identified TELO2 within the three segments of helical solenoids by obtaining a reconstruction from an R2-TT pull-down complex that lacks TELO2. While this was only of limited resolution (∼9 Å), density corresponding to much of the helical solenoids was evident (Figure S2). However, when the R2-TT map was compared with that for the R2-TTT complex, the segment in orange was not observed, even at a very low contour level, suggesting that it is due to the absence of TELO2 in the R2-TT complex. R2 does not contact TELO2 directly, which agrees with our observation that TELO2 is dispensable for the interaction of TT with R2 (Figure 1A).

**Figure 2:**
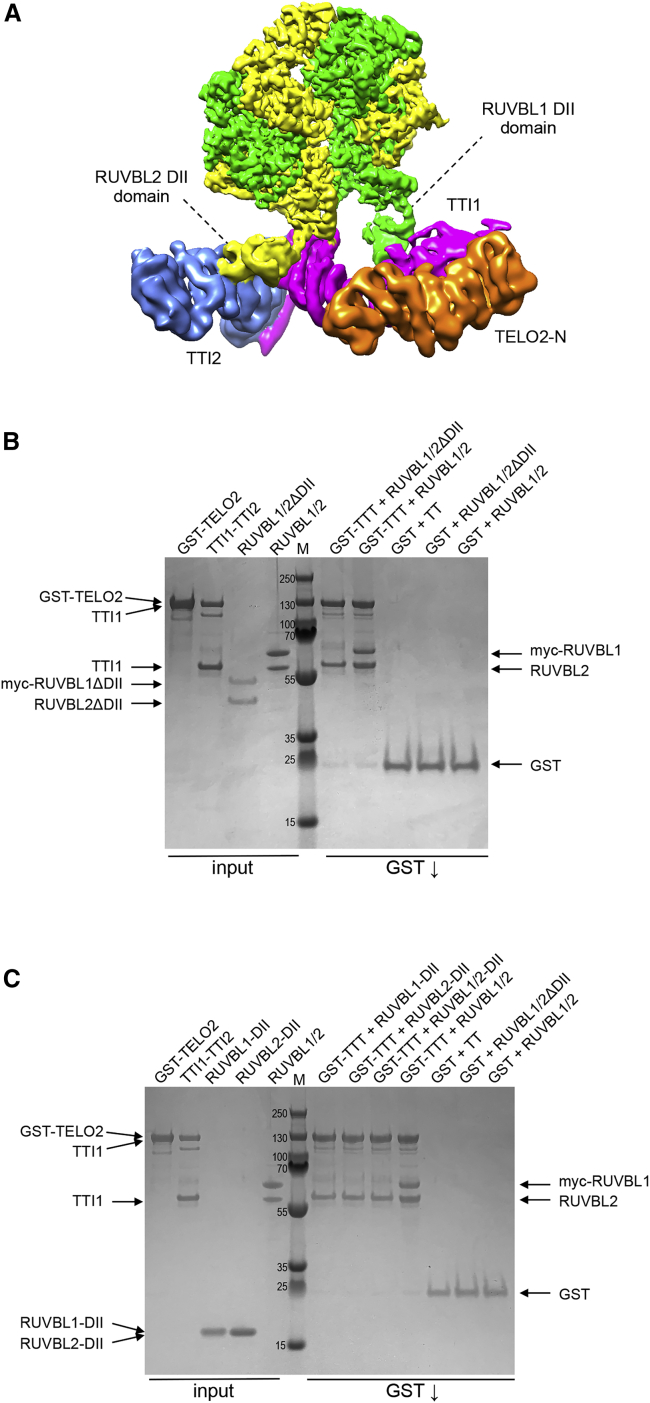
RUVBL1-RUVBL2 DII domains mediate TTT interaction (A) View of human R2-TTT volume colored as in Figure 1D, highlighting the contacts between the DII domains in two consecutive RUVBL subunits and the elongated TTT subcomplex. (B) Coomassie-stained SDS-PAGE gel showing analysis of dependence of the interaction of TTI1-TTI2-TELO2 (TTT) with RUVBL1-RUVBL2 on the RUVBL1/2 DII domains. Pull-down on the GST-tag attached to the N terminus of TELO2 co-precipitates TTI1-TTI2 and wild-type RUVBL1/2, but not the RUVBL1/2ΔDII complex where the DII domains have been excised. (C) As in (B), but showing that the isolated DII domains of RUVBL1 and RUVBL2, individually or in combination, are not co-precipitated by GST-TELO2-TTI1-TTI2 (GST-TTT) when not presented as part of the RUVBL1/2 heterohexamer.

The cryo-EM map revealed that TTT engages simultaneously with the DII domains from consecutive RUVBL1 and RUVBL2 subunits of the R2 ring (Figure 2A), primarily contacting the OB folds of the DII domains, which are themselves flexibly attached to the core of the ring. No direct contact between TTT and the highly ordered AAA+ ATPase core of the ring is evident. To test the importance of the DII domains in the recruitment of TTT, we generated an R2 complex from ΔDII mutants of each RUVBL subunit in which the antiparallel β strand connecting the DII domain to the ATPase core was truncated to a hairpin, thereby eliminating the pendant OB-fold region. This truncated R2 has been shown to oligomerize in the same way as the wild-type protein (Gorynia et al., 2011). While a glutathione *S*-transferase (GST)-tagged TTT complex co-precipitated wild-type R2 in pull-down experiments, it failed to co-precipitate the ΔDII R2, confirming that the OB folds of the DII domains are required for the interaction with TTT (Figure 2B). However, in the inverse experiment, the GST-tagged TTT was unable to effectively bind the RUVBL1 and RUVBL2 DII domains when isolated from the R2 complex of both subunits, either in pull-down experiments (Figure 2C) or size-exclusion chromatography (Figure S3). Taken together, these results show that the DII domains mediate the recruitment of TTT into the R2-TTT super-complex, but only when presented in combination within the defined spatial configuration of the heterohexameric R2 ring, as suggested by the cryo-EM structure.

### Atomic model of human TTT complex

**Figure 3:**
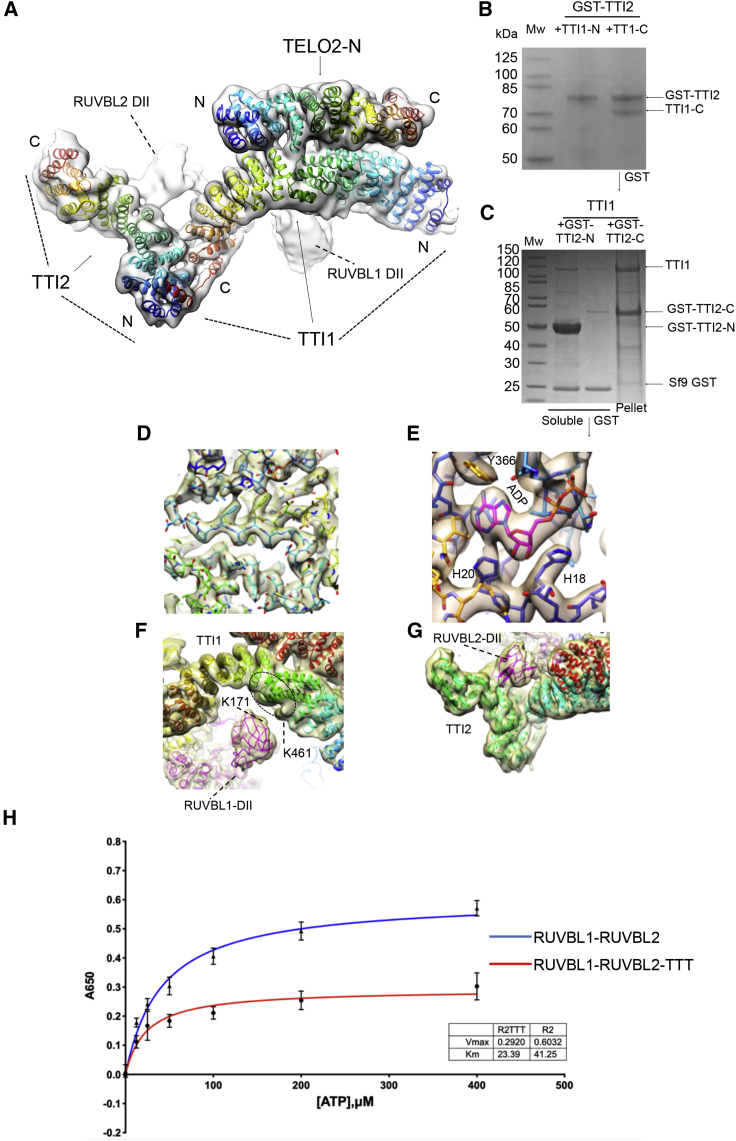
Molecular modeling of TTT and its interaction with RUVBL1-RUVBL2 (A) Molecular model of TTT fitted within the cryo-EM density of R2-TTT (transparent density). Individual helices of TTI1 and TTI2 were fitted manually into density using COOT and connected into a continuous structure by taking advantage of the repetitious linear right-handed α-solenoid architecture of HEAT repeats. The polarity of the two polypeptides was determined by the results of the interaction mapping experiments shown in (C) and (D). The fit of the poly-alanine structures was optimized by real-space refinement in Phenix (Adams et al., 2010). A homology model of the N-terminal domain of human TELO2 based on the crystal structure of *S. cerevisiae* Tel2p (Takai et al., 2010) was manually adjusted to fit the experimental density using COOT (Emsley and Cowtan, 2004). The TTT model was optimized by real-space refinement in Phenix (Adams et al., 2010). Each protein is colored as a rainbow from N- to C-terminal ends. (B) Co-expression of GST-tagged full-length TTI2 with TTI1 constructs spanning residues 1–459 (TTI1-N) and residues 468–1089 in Sf9 insect cells. Only the TTI1-C construct was co-precipitated as a soluble species with GST-TTI2, suggesting that TTI2 primarily interacts with the C-terminal half of TTI1. (C) Co-expression of full-length TTI1 with GST-tagged TTI2 constructs spanning residues 1–193 (TTI2-N) and residues 194–508 (TTI2-C) in Sf9 insect cells. The GST-tagged TTI2-N was well expressed as a soluble species and co-precipitated TTI1 in GST pull-downs, whereas co-expressed TTI1 and GST-TTI2-C were primarily found in the insoluble fraction. This suggests that TTI1 primarily interacts with the N-terminal half of TTI2. (D) Close-up of electron density in the core of the RUVBL2 subunit that contacts TTI1 and TTI2, showing clear electron density for side chains. The fit of the all-atom model of the RUVBL1-RUVBL2 heterohexamer was optimized by real-space refinement in Phenix. (E) Close-up of electron density in the nucleotide-binding site of the RUVBL1 subunit that contacts TTI1, showing bound ADP. All six nucleotide-binding sites in the R2 ring are fully occupied by ADP, and the His-Ser-His motif that closes off the nucleotide-binding site as part of the N-terminal “gatekeeper” segment (Munoz-Hernandez et al., 2019) is also fully ordered in all six ATPase subunits. (F) Interaction of TTI1 (rainbow-colored helices) with the DII domain of a RUVBL1 subunit (magenta). The fit of the all-atom model of the RUVBL1-RUVBL2 heterohexamer and the TELO2-N domain as well as the poly-Ala models of TTI1 and TTI2 were simultaneously optimized by real-space refinement in Phenix against a “consensus” electron density map comprising maps from separate multibody and focused refinements of the R2 rings and TTT with the interacting DII domains. In an XL-MS analysis of R2TP-TTT, a single cross-link was observed between RUVBL1-Lys171 (black circle) and Lys461 of TTI1. Although the resolution of the TTI1 density does not permit fitting of the sequence, the crosslink helps localize the approximate location of TTI1-Lys461 (red ellipse). (G) Interaction of TTI1 (cyan helices) and TTI2 (green helices) with the DII domain of a RUVBL2 subunit. The DII domain packs into the cleft formed at the junction of the α-helical solenoids of the two HEAT repeat proteins. (H) Human RUVBL1-RUVBL2 displays a weak inherent ATPase activity with a maximal rate of ~0.6 mol/min/mol. This is significantly diminished in the presence of TTT at a 3:1 molar ratio. Error bars indicate the standard error of the mean of three experiments.

We built an atomic model of TTT by assigning regions of HEAT repeat in the cryo-EM map and resolving uncertainties by mapping interactions between truncated versions of the TTT components. The crystal structure of an engineered construct of yeast Tel2p (Takai et al., 2010) consists of two helical solenoid segments of 19 and 9 helices, respectively, separated by ∼60 unstructured residues that include the phosphorylated binding motif for the PIH domain of Pih1p (Hořejší et al., 2014; Pal et al., 2014). The size and shape of the segment assigned to TELO2 in the cryo-EM structure (Figures 1D and 2A, orange) correspond well with the structure of the larger N-terminal segment of yeast Tel2p, and a homologous model of the corresponding region of human TELO2 could be readily docked into this density in the R2-TTT map as a rigid body (Figure 3A). The substantial interface between the TELO2 N-terminal domain (TELO2-N) and TTI1 is consistent with the identification of the N-terminal segment of Tel2p rather than the C-terminal segment as the main interactor with Tti1p-Tti2p (Takai et al., 2010). Based on this, we can then attribute the remaining segment of HEAT repeats to the ∼500 residues of TTI2. No significant density feature is evident that could correspond to the smaller C-terminal region of TELO2. As this region does not bind TTI1-TTI2 directly, and connects to the N-terminal domain by a long flexible linker containing the CK2 phosphorylation site implicated in interactions with the PIH domain of PIH1D1, we conclude that it is disordered in the present structure.

No prior structures are available for TTI1 or TTI2 or homologs thereof, intact or in part. While the local resolution of the maps in these regions (4.5–5Å) is sufficient to define the positions and topological connection of the α helices making up the HEAT repeats that constitute most of these two proteins, it is not high enough to allow assignment of amino sequence or the orientation of the Cα-Cβ vectors of the side chains, which indicates the N→C polarity of the helix. Consequently, the direction of the polypeptide chain in the α-helical solenoid cannot be directly determined.

To solve this problem, we took advantage of the effectively linear mapping of primary to tertiary structure in α-helical solenoid architectures such as the HEAT repeats that are predicted to form the majority of TTI1 and TTI2. One end of the helical density ascribed to TTI2 comes into close contact with one end of the long arc of helical density ascribed to TTI1. To determine which end of the TTI1 sequence this corresponds to, we co-expressed separate strep-tagged N-terminal (1–459) and C-terminal (468–1089) parts of TTI1 with GST-tagged full-length TTI2 in insect cells. Strep-TTI1-C was clearly recovered along with GST-TTI2 in glutathione bead pull-downs from TTI1-C-expressing cells, whereas no strep-TTI1-N was observed in similar experiments with the TTI1-N-expressing cells (Figure 3B). Correspondingly, GST-TTI2 was co-precipitated with strep-TTI1-C, but neither strep-TTI1-N nor GST-TTI2 was recovered by strep-tag pull-down from strep-TTI-N-expressing cells (Figure S4). In a similar experiment we co-expressed full-length TTI1 in insect cells, with either the N-terminal (1–193) or C-terminal (194–508) part of TTI2 as a C-terminal fusion to GST. We found that the GST-TTI2-N was highly expressed as a soluble protein, and it co-purified full-length untagged TTI1 in glutathione bead pull-downs. In contrast, the GST-TTI2-C fusion was barely detectable in the soluble fraction and did not co-precipitate TTI1 (Figure 3C). Taken together, these results indicate that the TTI1-TTI2 interface involves the N terminus of TTI2 binding to the C terminus of TTI1 with the more central/N-terminal part of TTI1 providing the binding site(s) for the TELO2 N-terminal domain (Figure 3A). We were then able to dock known structures or homology models of RUVBL1, RUVBL2, TELO2-N, and the helical repeats of TTI1 and TTI2 into the cryo-EM maps and optimize the atomic fits using restrained real-space refinement in phenix.refine (Adams et al., 2010) and manual adjustment in COOT (Casanal et al., 2020).

### RUVBL1-RUVBL2–TTT Interactions regulate ATPase activity

The structure of the RUVBL1-RUVBL2 ring (maximum resolution 3.41Å) within the overall human complex is similar to that previously described in the R2TP complex (Martino et al., 2018; Munoz-Hernandez et al., 2019).The amino acid sequence of all six subunits is well resolved with clear side-chain density evident for most of the polypeptide chain (Figure 3D), apart from the DII insertion domains. Two of these, which interact with the TTT proteins, are resolved at resolutions in the 4.5–7.5 Å range, while the other four are substantially disordered. All six subunits have clear density for bound ADP, with the N-terminal “gatekeeper” segment of polypeptide incorporating the His-Ser-His motif, well ordered and interacting with the bound nucleotide (Munoz-Hernandez et al., 2019) (Figure 3E).

Although recruitment of TELO2 (Tel2p in yeast), and by presumption the entire TTT subcomplex, to R2TP was thought to depend on interaction of a conserved CK2 phosphorylation site in TELO2/Tel2p with the PIH domain of PIH1D1/Pih1p (Hořejší et al., 2014; Horejsí et al., 2010; Pal et al., 2014), our 3D structures and supporting biochemistry clearly show substantial direct interactions between elements of the TTT subcomplex and the RUVBL ring that are completely independent of the TP components—RPAP3/Tah1p and PIH1D1/Pih1p—and are sufficient to form stable R2-TTT and R2-TT complexes.

At the heart of the TTT interface with R2, the concave inner surface of TTI1 wraps around the OB fold of the projecting DII domain of a RUVBL1 subunit, which contacts helices from the central part of the TTI1 solenoid, on the face opposite to the binding site of the TELO2-N segment (Figure 3F). Consistent with this arrangement, we identified a single cross-link between RUVBL1 and TTI1 in a cross-linking mass spectrometry (XL-MS) analysis of a putative human R2TP-TTT complex (Figure S4), connecting Lys171 of RUVBL1 with Lys461 of TTI1. While the resolution of the TTI1 density does not allow this residue to be pinpointed with any precision, it does localize it to a region encompassing one or two helical segments in the fitted model and helps establish the approximate staging of the sequence along that model. Simultaneously, the DII domain of the clockwise adjacent RUVBL2 subunit packs into the V-shaped cleft formed at the junction of the predicted C terminus of TTI1 and the predicted N terminus of TTI2 (see above), interacting with helices from the convex faces of both proteins (Figure 3G). As essentially all the contacts between the TTT subcomplex and the R2 ring are mediated by the DII domains, which are themselves very flexibly attached to the AAA+ ATPase core, there is substantial conformational variability in the relative orientation of the TTT and R2 components as revealed by multi-body refinement (Nakane et al., 2018).

We previously showed a direct coupling between the conformation of an N-terminal region in RUVBL2 containing the His-Ser-His motif that binds nucleotide (Figure 3E) and the conformation of DII domains, which was promoted by their interaction with PIH1D1 (Munoz-Hernandez et al., 2019). This was shown to affect the binding of nucleotides to regulate nucleotide exchange as part of the AAA+ ATPase cycle. As the TTT complex makes extensive interaction with the DII domains of a pair of consecutive RUVBL1 and RUVBL2 proteins in the heterohexameric ring, we explored whether TTT binding influenced the AAA+ ATPase cycle. Using a sensitive malachite green phosphate-release assay, we were able to measure a basal ATPase activity with V_max_ ∼0.6 mol/min/mol for human RUVBL1-RUVBL2, which was substantially diminished by addition of a 3:1 molar equivalent of TTT (Figure 3H). Therefore, TTT directly binds RUVBL and modulates its ATPase activity.

### PIH1D1/Pih1p and TTT compete for the interaction with RUVBL1-RUVBL2

TTT is assumed to function in the context of R2TP, a complex where R2 also binds RPAP3-PIH1D1 (TP). As we have previously shown that PIH1D1 also interacts with the DII domains of R2, we explored whether TTT and PIH1D1 compete for interaction with R2 or can be accommodated together within a super-complex. For this, we assembled the human R2TP-TTT complex for cryo-EM studies by mixing separately purified R2TP and TTT complexes and the mix was analyzed by cryo-EM.

**Figure 4:**
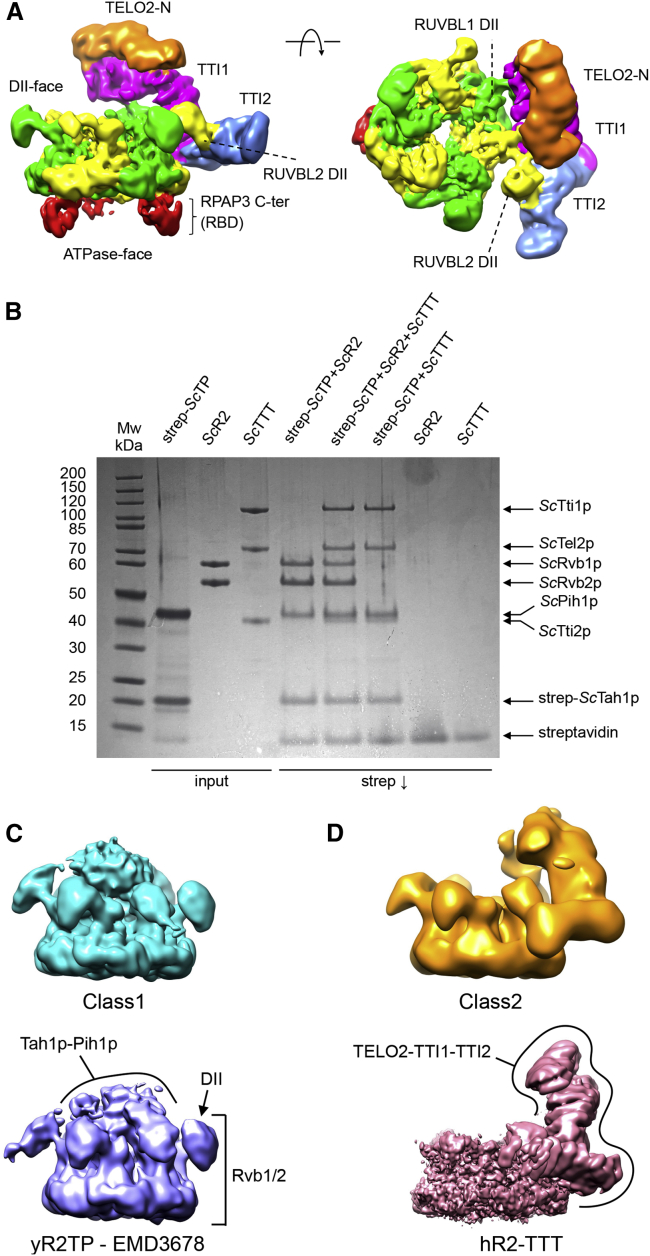
Cryo-EM of human and yeast R2TP-TTT complexes (A) Two views of human R2TP-TTT volume: RUVBL1-RUVBL2 hexamer, green and yellow respectively; TTI1, magenta; TTI2, blue; TELO2-N, orange. The RPAP3 C-terminal RUVBL2-binding domain (RBD) is identifiable in the ATPase face of each of the RUVBL2 subunits (red). (B) Coomassie-stained SDS-PAGE gel showing analysis of interaction of yeast Tah1p-Pih1p (yTP), Rvb1p-Rvb2p (yR2), and Tel2p-Tti1p-Tti2p (yTTT) subcomplexes. Pull-down on the tandem strep-tag attached to the N terminus of Tah1p within the yTP complex co-precipitates yR2 and yTTT simultaneously and as separate co-complexes. (C) Cryo-EM analysis of co-precipitated Tah1p-Pih1p-Rvb1p-Rvb2p-Tel2p-Tti1p-Tti2p (yeast R2TP-TTT). 3D classification yields two main classes: one class contained particles with strong central density between the DII insertion domains of the R2 ring comprising two-thirds of the particles (top), and the other class resembled the previously described yeast R2TP complex (EMD-3678) (Rivera-Calzada et al., 2017) (bottom). (D) As in (C), but showing the second main class comprising two-thirds of the particles, which lack the central density attributed to Tah1p-Pih1p, but display strong peripheral density at one edge of the R2 ring (top) that strongly resembles the density for the TTT complex in the higher resolution human R2TTT structure (bottom). No significant class of particles was identified in which the central and peripheral density features were simultaneously present.

We obtained a reconstruction for the R2TP-TTT complex with an average resolution of 6.1 Å in which, as well as a bound TTT subcomplex, we could readily identify two well-defined copies of the C-terminal RBD of RPAP3 bound to the AAA face of the RUVBL1-RUVBL2 ring (Martino et al., 2018; Maurizy et al., 2018) (Figure 4A; Figure S5). As with previous studies, a third RBD was evident at a lower contour level. The structural organization of TTT in R2TP-TTT was essentially identical to that observed in the R2-TTT complex. In R2TP, whereas three RPAP3-PIH1D1 complexes can be tethered to each RUVBL1-RUVBL2 ring at a time due to the interaction of the RPAP3-RBD domain to each of the three RUVBL2 subunits, only one PIH1D1 is effectively engaged to the DII domains (Martino et al., 2018; Munoz-Hernandez et al., 2019). Interestingly, although present in the *in vitro* assembled R2TP-TTT complex, no density was evident for PIH1D1 at the binding site identified in previous studies, nor for the conformationally flexible N-terminal regions of RPAP3 (Martino et al., 2018; Munoz-Hernandez et al., 2019). This suggests that TTT and PIH1D1 might compete for the binding to the DII domains of R2.

To further support this hypothesis, we also assembled an *S. cerevisiae* R2TP-TTT complex *in vitro* by mixing a pre-assembled Rvb1p-Rvb2p-Pih1p-Tah1p complex with an equal concentration of a pre-assembled Tel2p-Tti1p-Tti2p complex where the components were purified from insect cells to ensure substantial post-translational modifications on the Tel2p component. Yeast TTT prepared in this way was co-precipitated in pull-down experiments with Tah1p-Pih1p (Figure 4B), suggesting that any phosphorylation required within the Tel2p motif predicted to bind the PIH domain of Pih1p was present on the sample. Cryo-EM data were collected and processed as above. 3D classification of particles from this sample revealed a more heterogeneous distribution than for the human samples, which ultimately partitioned into two main classes. The more populated and higher resolution (∼6 Å) class possessed a strong centrally positioned feature nestled between the DII domains of the R2 ring, but with no density corresponding to TTT, and it strongly resembled the previously described yeast Rvb1p-Rvb2p-Pih1p-Tah1p complex structure (Rivera-Calzada et al., 2017) (Figure 4C). The second, less populated and lower resolution class (∼10 Å) lacked the central feature, but it possessed a substantial additional density at the periphery of the R2 ring, which resembled the human TTT density in the human R2TTT reconstruction, and in which similar segments could be identified (Figure 4D). No significant classes were found in 3D classification in which the central and peripheral density features were simultaneously present.

Therefore, our analysis of human and yeast R2TP bound to TTT suggests that TTT and PIH1D1 compete for their interaction with the DII domains.

### TTT delivers TOR to the R2TP chaperone

**Figure 5:**
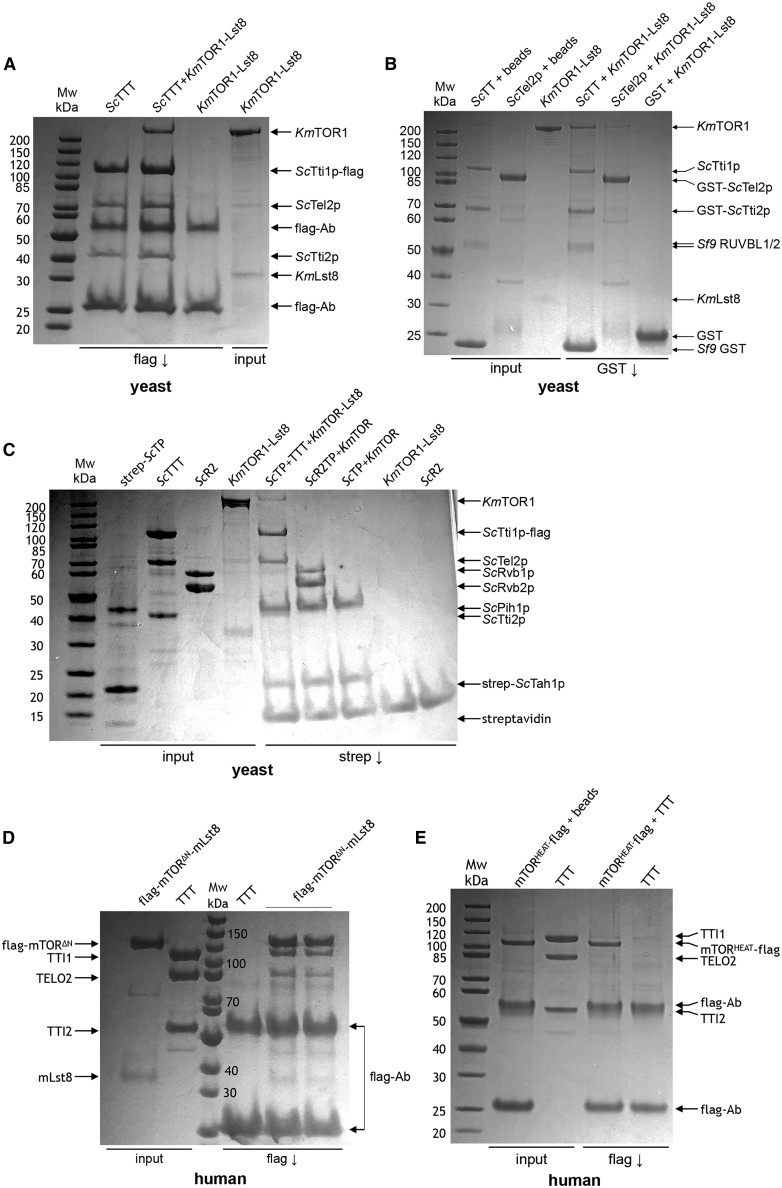
Mapping the interactions between TTT and TOR (A) Coomassie-stained SDS-PAGE gel showing interaction of yeast (*Kluyveromyces marxianus*) TOR1-Lst8 with yeast (*S. cerevisiae*) TTT. FLAG-tagged Tti1p co-immunoprecipitates TOR1, whereas anti-FLAG beads alone do not. Lst8, which is evident as a weakly staining band in the input, was not evident in the immunoprecipitated TTT-TOR1. (B) As in (A), but for interaction of KmTOR-Lst8 with GST-tagged Tti2p-Tti1p or GST-tagged Tel2p. KmTOR was co-precipitated in GST pull-down with Tti1p-GST-TTI2p, but not with Tel2p. (C) As in (A), but for interaction of KmTOR-Lst8 with TP and R2TP complexes containing strep-tagged Tah1p. Neither TP nor R2TP complexes were able to co-precipitate KmTOR directly. However, tagged TP could co-precipitate KmTOR when TTT was present, confirming that TP and KmTOR do not compete for binding to TTT. (D) As in (A), but for interaction of a FLAG-tagged N-terminally truncated human mTOR construct (mTOR^ΔN^) co-expressed with mLST8, with human TTT. TTT is co-immunoprecipitated by FLAG-tagged mTOR^ΔN^, but not by FLAG beads alone. (E) As in (A), but for interaction of a FLAG-tagged soluble N-terminal fragment of human mTOR encompassing the major segment of HEAT repeats (mTOR^HEAT^) with human TTT. Flag-tagged mTOR^HEAT^ was precipitated by FLAG beads, but it did not co-immunoprecipitate TTT.

While our data suggest that TTT also plays a role in regulating the ATPase activity of the R2 ring, its main function is thought to be as an adaptor, recruiting PIKK client proteins such as TOR to the R2TP or R2 complex (Houry et al., 2018; Kakihara and Houry, 2012; Muñoz-Hernández et al., 2018). To test this, we purified *S. cerevisiae* TTT in which Tti1p carried a C-terminal FLAG-tag and looked at its ability to interact with a TOR1-Lst8 complex from the closely related yeast *Kluyveromyces marxianus* (the construct was a kind gift of Roger Williams, MRC-LMB, Cambridge, UK), which has 83% sequence similarity to *S. cerevisiae* TOR2, but is far more amenable to large-scale expression and purification. We found that KmTOR was co-immunoprecipitated by beads carrying an anti-FLAG antibody in the presence of FLAG-tagged TTT, but not in its absence (Figure 5A). KmLst8 was evident in the input TOR1 preparation as a weak-staining band. Curiously, this band was less visible in the immunoprecipitated material, but since Lst8 in our preparations is always much weaker staining in gels than its TOR partner, we cannot conclude whether this reflects some effect of TTT in the interaction between TOR and Lst8. FLAG-tagged TTT was also able to simultaneously co-immunoprecipitate KmTOR and R2 when both components were present, suggesting that KmTOR, R2, and TTT can form a complex (Figure S6). To determine which components of the TTT complex are required for interaction with the PIKK client, we separately expressed Tel2p and the constitutive Tti1p-Tti2p complex and looked at their interaction with KmTOR. We found that KmTOR was effectively co-precipitated with Tti1p - GST-Tti2p in a GST pull-down experiment, but not by GST-Tel2p (Figure 5B). In contrast to TTT, TP (Tah1p-Pih1p) or R2TP complexes in which Tah1p carried a tandem strep-tag were unable to co-precipitate KmTOR directly. However, strep-tagged TP could co-precipitate KmTOR when TTT was also present, demonstrating that TTT can provide a bridging interaction between the two, in which the binding sites for TP and KmTOR on TTT are non-overlapping (Figure 5C).

**Figure 6:**
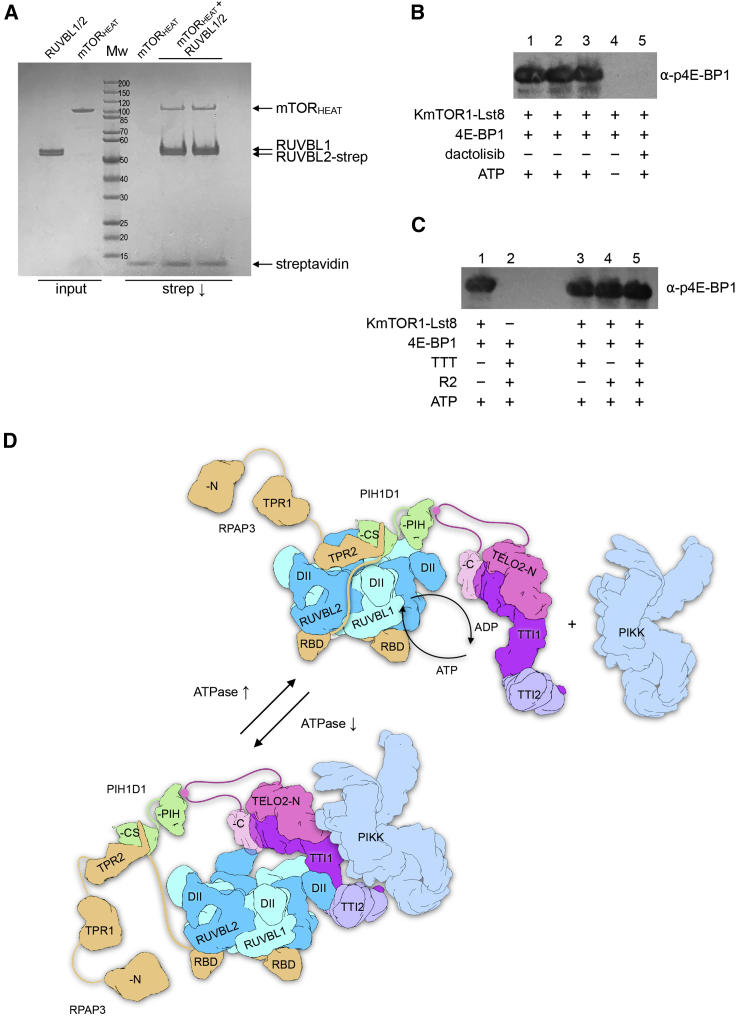
TTT couples mTOR recruitment to R2TP to regulation of the chaperone (A) Coomassie-stained SDS-PAGE gel showing the interaction of an mTOR fragment comprising the N-terminal HEAT region (residues 1–929) with the strep-tagged RUVBL1-RUVBL2 complex. (B) Western blot showing phosphorylation of 4E-BP1 by the yeast (*K. marxianus*) TOR1-Lst8 complex. A strong signal was detected by a phosphospecific antibody to pThr37/46 in the presence of ATP, but not when ATP was omitted from the reaction, or when the ATP-competitive mTOR inhibitor dactolisib (NVP-BEZ235) was present. (C) As in (B), but with the addition of TTT, R2, or both. Neither of these chaperone components, alone or in combination, affects its ability to phosphorylate a substrate. (D) Model of the functions of TTT. Flexible tethering to the R2 ring allows for facile exchange of TP and TTT components at the main interaction site on the DII domain face of the ring. Interaction of the PIH1D1 part of TP with a RUVBL1 DII domain facilitates partial ring opening and nucleotide exchange (Munoz-Hernandez et al., 2019), accelerating the basal ATPase activity of the ring (Rivera-Calzada et al., 2017). The CK2 phosphorylation site within the inherently disordered linker connecting the N- and C-terminal domains of TELO2 (Horejsí et al., 2010) tethers the TTT subcomplex to the R2 ring through interaction with the PIH domain of PIH1D1 (Hořejší et al., 2014; Pal et al., 2014) and thereby facilitates recruitment of a PIKK client to the core complex (top). Rearrangement of the complex allows TTT to bind to the DII domains of consecutive RUVBL1-RUVBL2 domains, fixing them in an ADP-bound closed state that downregulates the basal ATPase, potentially bringing the PIKK client into closer association. Steric hindrance of the DII domains, as well as allosteric incompatibility, prevents simultaneous binding of PIH1D1 to the DII face of the ring. Although not engaged, TP remains tethered to the complex through the interaction of the PIH domain with the TELO2 linker segment (see above) and the interaction of the C-terminal domain of RPAP3 with the ATPase face of the R2 ring (bottom).

We also expressed and purified a FLAG-tagged construct of human mTOR (mTOR^ΔN^) that lacks most of the N terminus (residues 1–1376) but retains the binding site for mLst8, and it displays essentially full kinase activity (Yang et al., 2013).We found that the human TTT complex was co-immunoprecipitated by beads carrying an anti-FLAG antibody, in the presence of a FLAG-tagged mTOR^ΔN^-mLst8 complex, but not in its absence (Figure 5C). We also expressed and purified a soluble FLAG-tagged segment of mTOR comprising the first 929 residues. We found that this construct, which encompasses the main HEAT repeat segment of mTOR, was not able to co-precipitate human TTT (Figure 5E); however, this fragment was able to interact with RUVBL1-RUVBL2 in the absence of TTT (Figure 6A).

Taken together, these data suggest that RUVBL1-RUVBL2 and TTT cooperate to bring TOR to the complex. The binding site for TTT on TOR actually resides in the C-terminal kinase region, rather than in the N-terminal HEAT repeat region, as had previously been suggested (Takai et al., 2007), and it primarily involves a substantial direct contribution from TTI1/Tti1p-TTI2-Tti2p rather than TELO2/Tel2p itself (Takai et al., 2010). RUVBL1-RUVBL2 instead interacts with the mTOR N-terminal HEAT repeat region.

Since TTT and R2 form direct complexes with mTOR, we sought to determine whether these interactions affected the ability of TOR to phosphorylate its substrates. We used a phosphospecific antibody to a highly conserved and well-characterized TOR target phosphorylation site in 4E-BP1 (Wu et al., 2017) to show that our purified yeast KmTOR1-Lst8 was active as a kinase (Figure 6B) and was effectively inhibited by an ATP-competitive mTOR inhibitor (Liu et al., 2009). However, we found that addition of an excess of TTT or R2, or both together, had no effect on the ability of KmTOR1 to phosphorylate 4E-BP1 (Figure 6C), suggesting that the interactions that these components of the R2TTT complex make with TOR proteins do not have a role in regulating TOR kinase activity directly.

## Discussion

The heterohexameric RUVBL1-RUVBL2 ring appears to provide the central constant component of a multiplicity of chaperone complexes each specific for a distinct class of client proteins (Houry et al., 2018; Kakihara and Houry, 2012; Muñoz-Hernández et al., 2018). As with systems such as HSP90, with which RUVBL1-RUVBL2 (R2) collaborates as a co-chaperone, client specificity is thought to be mediated by scaffold proteins that structurally couple the generalized core R2-ATPase complex to the client. In both yeast and mammalian systems, the TELO2-TTI1-TTI2/Tel2p-Tti1p-Tti2p (TTT) complex, whose structure we describe herein, behaves as a classical scaffold adaptor, being able to independently bind a PIKK protein and the RUVBL1-RUVBL2 ring (Figures 2 and 5).

Current models suggest that recruitment of TTT to R2 is mediated by the interaction of a conserved phosphopeptide motif within the intrinsically disordered linker segment of TELO2/Tel2p, which binds to the PIH domain of PIH1D1/Pih1p (Hořejší et al., 2014; Horejsí et al., 2010; Pal et al., 2014) in the TPR protein-PIH (TP) component of an R2TP-TTT super-complex. However, our structural and biochemical data clearly show that human TTT is fully able to bind R2 in the absence of PIH1D1 and RPAP3 (Figure 1), so while this interaction may contribute to the function of the system, it is not essential for TTT recruitment. In the human system, the TTT and TP subcomplexes are able to coexist simultaneously in a complex with the same R2 ring, as demonstrated by the clear presence of density for RPAP3-RBD domains on the ATPase face of the R2 ring to TTT in cryo-EM reconstructions (Figure 4A). However, no density was evident that would correspond to the expected location, based on our previous structural analysis (Munoz-Hernandez et al., 2019) of PIH1D1 attached to one of those RPAP3 molecules and engaged with the DII side of the R2 ring. The absence of density for PIH1D1, although present in the complex through its constitutive association with RPAP3, suggests that binding of TTT and PIH1D1 to the DII face of the R2 ring may be mutually exclusive.

In the low-resolution yeast cryo-EM data, we identify only two classes of particles, in which either TP or TTT subcomplexes, but not both, are bound to the DII face of the R2 ring (Figures 4C and 4D). The absence of any substantial set of particles displaying density for both TP and TTT components implies that as in the human system, binding of these components to the R2 ring is mutually exclusive.

Taken together, our observations for human and yeast systems suggest that TTT and TP components are alternative R2 ligands engaging with the DII face of the ring with mutual exclusivity. However, the independent pairwise interactions of the TTT, TP, and R2 components keep the non-engaged component tethered to the overall complex. In the yeast system, this tethering is provided by binding of the PIH domain of Pih1p in the TP component to a putative CK2 phosphorylation site within an intrinsically disordered segment of ∼50 residues that links the N-terminal and C-terminal domains of Tel2p. In the human system, in addition to the equivalent interaction of the PIH1D1 PIH domain and the CK2 phosphorylation site in the longer ∼70-residue TELO2 linker segment, the TP component is also tethered by the C-terminal RBD domain of RPAP3, which connects by an intrinsically disordered segment of ∼100 residues to the binding motif of for the CS domain (Henri et al., 2018) (Figure 6D).

This flexible tethering mechanism might serve to allow the non-engaged component to nonetheless remain associated with the overall complex and poised to exchange with the engaged component as required. We previously showed that binding of the TP component accelerated the inherent ATPase of the R2 ring (Rivera-Calzada et al., 2017), probably by promoting ring opening to facilitate nucleotide exchange (Munoz-Hernandez et al., 2019). In this study, we show that binding of the TTT complex has the converse effect, decreasing ATP turnover by stabilizing the ring in a closed ADP-bound state. The flexible exchange of TP and TTT subcomplexes might therefore function in regulating the ATPase activity of the system at different stages of loading, manipulation, and release of a PIKK client protein; however, further work is required to test this idea.

TELO2/Tel2p and the associated TTI1/Tti1p-TTI2/Tti2p are implicated in the activation and stabilization of most, if not all, members of the PI3K-like kinase family (Takai et al., 2007). These proteins share strongly conserved C-terminal kinase regions attached to more diverse N-terminal segments composed of HEAT repeats. Paradoxically, a previous cell-based interaction analysis using expressed fragments of mTOR or ATM implicated segments of the less conserved HEAT repeats in the interaction with TELO2 (Takai et al., 2007). Using purified and soluble segments of mTOR, we find instead that it is the strongly conserved kinase-containing C-terminal region that is recognized by the TTT complex. However, this interaction does not inhibit kinase activity, indicating that it neither allosterically regulates TOR nor prevents access of substrate to its active site (Figure 6C). While we cannot dismiss the possibility that parts of the HEAT repeats of particular PIKKs may contribute to their individual interaction with TTT, the primary involvement of the common and highly conserved kinase region provides a more evolutionarily compelling explanation for the selectivity of the TTT component of this chaperone/co-chaperone system. Nonetheless, RUVBL1-RUVBL2 binds independently of TTT to the N-terminal HEAT regions in mTOR, suggesting that both TTT and R2 components collaborate to mediate PIKK interactions.

Taken together, our results reveal the architecture of the TTT complex and show how TTT couples R2TP’s recruitment of PIKKs to the regulation of the chaperone’s ATPase activity. The structural details of the interaction of TTT and its complexes with PIKKs and their complexes remain to be determined. However, the ability of TTT to interact with fully folded and catalytically competent mTOR is consistent with previous observations that interaction with TTT is not required for folding, but instead contributes to the assembly of the larger multiprotein complexes in which PIKKs such as mTOR are key components (Takai et al., 2010). Further work is required to uncover how R2TP-TTT facilitates that process.

## STAR★Methods

### Key resources table

**Table:** 

REAGENT or RESOURCE	SOURCE	IDENTIFIER
**Bacterial and virus strains**		

*E.coli* BL21 (DE3) *pLysS*	Thermo Fisher Scientific	Cat# 10604973
*E.coli* DH5α	Thermo Fisher Scientific	Cat# 11583117
E.coli DH10Bac	Thermo Fisher Scientific	Cat# 10361-012

**Chemicals, peptides, and recombinant proteins**		

DSS-H_12_/D_12_	Creative Molecules Inc	Cat# 001S
Trypsin	Promega, UK	Cat# V5111
Glu-C protease	Promega, UK	Cat# V1651
2XFlag-peptide	Peptide synthetics, UK	Quote Q23477
L-Glutathione reduced	Thermo Fisher Scientific	Cat# 10628343
ANTI-FLAG® M2 Affinity Gel	Sigma-Aldrich	Cat# A2220
Complete, EDTA-free protease inhibitor tablets	Roche Diagnostics	Cat# 05056489001
PhosSTOP	Sigma-Aldrich	Cat# 4906845001
PreScission and TEV protease	University of Sussex	N/A
Phospho-4EBP1 (Thr37/46) polyclonal antibody	Cell Signaling Technology	Cat# 236B4

**Critical commercial assays**		

P_i_ColorLock Gold Phosphate Detection System	Innova Biosciences	Cat# 303-0030
Cellfectin II Reagent	Thermo Fisher Scientific	Cat# 10362100

**Deposited data**		

R2TP-TTT cryoEM volume	this work	Electron Microcopy Databank (EMDB): EMD:12957
R2-TTT cryoEM volume	this work	Electron Microcopy Databank (EMDB): EMD:12979
Atomic Coordinates for R2-TTT	this work	Protein Databank (PDB): PDB:7OLE

**Experimental models: Cell lines**		

Sf9 cells	Thermo Fisher Scientific	Cat# 11496015

**Recombinant DNA**		

P3E plasmid	University of Sussex	N/A
pFBDM plasmid expressing human and yeast TTT and mTOR-KD proteins	This study	N/A
KmTOR-KmLst8	A Gift from Prof. Roger Williams	N/A

**Software and algorithms**		

PRISM 8.0	GraphPad	https://www.graphpad.com/scientific-software/prism/
Relion-3.1	Zivanov et al., 2018	https://www2.mrc-lmb.cam.ac.uk/groups/scheres/impact.html
EPU software	Thermo Fisher Scientific	https://www.fei.com/software/epu-automated-single-particles-software-for-life-sciences/
MotionCor2	Zheng et al.*,* 2017	https://msg.ucsf.edu/software
GCTF and CTFFIMND4	Zhang, 2016; Rohou and Grigorieff, 2015	https://www2.mrc-lmb.cam.ac.uk/research/locally-developed-software/zhang-software/#gctf and https://grigoriefflab.umassmed.edu/ctf_estimation_ctffind_ctftilt
Phenix	Adams et al., 2010	https://www.phenix-online.org/
Coot	Emsley and Cowtan, 2004	https://www2.mrc-lmb.cam.ac.uk/personal/pemsley/coot
Pymol	Schrödinger	https://pymol.org/2/
Chimera	Pettersen et al., 2004	http://www.rbvi.ucsf.edu/chimera
I-TASSER	Yang et al., 2015	https://zhanglab.ccmb.med.umich.edu/I-TASSER/
Phyre^2^	Kelley et al., 2015	http://www.sbg.bio.ic.ac.uk/∼phyre2/html/page.cgi?id=index

**Other**		
Quantifoil 1.2/1.3, 300 mesh copper	Agar Scientific	Cat# AGS143-2
FEI-Vitrobot MAG IV	Thermo Fisher Scientific	N/A
Leica EM GP2	Leica microsystems	N/A
Titan Krios	Thermo Fisher Scientific	N/A
K2 direct detector camera	GATAN	N/A

### Resource availability

#### Lead contact

Further information and requests for resources and reagents should be direct to and will be fulfilled by the Lead Contact, LHP Laurence.pearl@sussex.ac.uk

#### Materials availability

Plasmids and constructs generated in this study can be requested from the lead contact.

#### Data and code availability

Real space-refined atomic coordinates for the R2-TTT model have been deposited in the Protein Databank (PDB) with accessions code: PDB: 7OLE. The composite cryoEM volume for R2TP-TTT and R2-TTT has been deposited in the Electron Microcopy Databank (EMDB) with accession code: EMD:12957 and EMD:12979 respectively.

### Experimental model and subject details

#### Bacterial strains and growth conditions

The bacterial cells, BL21 (DE3) pLysS (cat# 10604973), DH5α (cat# 11583117) and DH10Bac (cat# 10361-012) were purchased from Thermo Fisher Scientific. For protein expression, BL21 (DE3) pLysS cells were transformed with plasmids encoding different genes of the R2TP complex. The transformed cells were spread on LB agar plates containing ampicillin and kanamycin antibiotics. The plates were incubated at 37°C overnight, and a single colony of these transformed cells was used to grow starter culture for protein expression at a large scale (10L). The cells were grown in LB media containing ampicillin and kanamycin antibiotics. For future protein expression, these transformed cells were stored as 40% glycerol stocks at −80°C.

#### Virus and its propagation

Bac-to-Bac system was used to generate the baculoviruses, which were used to infect Sf9 cells. The bacmid DNA samples were stored at −20°C. The propagated baculoviruses were stored at 4°C supplemented with 2% Foetal Calf Serum (Sigma-Aldrich cat # 12133C).

#### *Spodoptera frugiperda* 9 (Sf9) cell growth conditions

For the insect cell protein expression, Sf9 cells (Spodoptera frugiperda 9) were purchased from Thermo Fisher Scientific (cat# 11496015). Sf9 cell culture of 4 × 105 cells/ml was added to the 500ml of Insect-Xpress media containing Streptomycin/Penicillin (Lonza) antibiotics in a 2L roller bottle. The cell culture was incubated at 27°C with 150rpm until the cell density reached 2x 106 cells/ml. Then the cells were infected with titered viruses expressing proteins of interest. The infected cells were further grown for 72h at 27°C using 150rpm rotation. The cells were pelleted by spinning 2000 x *g* (rotor JLA 8.1) for 10min, at 4°C. The cell pellet was frozen at −80°C for long term storage.

#### Yeast cell growth conditions

The Kluveromyces marxianus yeast strain (ATCC22296, LGC Standards Ltd., UK) expressing KmTOR1-KmLst8 was a gift from Dr Roger Williams (LMB, University of Cambridge). The cells were grown in yeast extract peptone (YEP) media supplemented with 2% glucose at 37°C and 220rpm. The protein expression and purification were carried out according to the published protocol by Dr Roger Williams’ lab (Baretić et al., 2016). The K. marxianus strain was stored as 40% glycerol stock at −80°C.

### Method details

The bacterial strain BL21 (DE3) *pLysS* was used to express yeast Tel2p proteins. The human and yeast TTT (Tel2-TTI1-TTI2) protein complexes, and human mTOR^N^ and mTOR^ΔN^-Lst8 were expressed in Sf9 cells. The *Kluveromyces marxianus* yeast was used to express full-length KmTOR1-Lst8 complex.

#### Gene cloning and protein expression and purification

Yeast (*Saccharomyces cerevisiae*) and human R2 (RUVBL1/2 or Rvb1/2) and TP (RPAP3-PIH1D1 or Tah1p-Pih1p) were expressed and purified as previously described (Martino et al., 2018; Rivera-Calzada et al., 2017). *Kluveromyces marxianus* KmTOR-KmLst8 were purified as described in (Baretić et al., 2016). Human and yeast TELO2/Tel2p were cloned into pFBDM plasmid as BamHI and EcoRI fragments, respectively, in fusion with a GST-tag. The yeast Tel2p was also cloned into p3E (Sussex University) using NdeI and BamHI fragment resulting into GST-Tel2p which was co-overexpressed with CKII (Sussex University) in BL21 (DE3) *pLysS* cells (Thermo Fisher Scientific). TTI1 and GST-TTI2 were cloned into pFBDM plamid as XhoI-KpnI and BamHI-EcoRI fragments, respectively (Genscript). Strep-TTI1_1-459_ and Strep-TTI1_468-1089_ were cloned into pFBDM-GST-TTI2 plasmid for coexpression. GST-TTI2_1-193_ and GST-TTI2_194-508_ and GST- mTOR^N^ (residues 1-929) were cloned into BamHI and EcoRI in pFBDM-TTI1 plasmid for *Spodoptera frugiperda* (Sf9) expression. The mTOR^ΔN^ (residues 1376 to 2549) containing 6XHis-2XFlag-PreScission cleavable tag at its 5′ was purchased from Genscript and cloned into BamHI and EcoRI sites of pFBDM and 5′ 2XFlag-6XHis-PreScission cleavable tagged-mLst8 gene was cloned as XhoI and KpnI fragment into pFBDM plasmid. TTI1-TTI2, full length as well as truncated versions and mTOR^ΔN^, mTOR^N^ complexes were overexpressed in Sf9 cells. For the co-expression of the yeast and human TTT complex, mixed viruses of yeast and human TTI1-TTI2/Tti1p-Tti2p and TELO2/Tel2p were used to co-infect Sf9 cells. Cell pellets containing yeast Tel2p were disrupted by sonication, while those containing TTI1-2 complex were lysed using a cell homogenizer in 20 mM HEPES pH 7.5, 250 mM NaCl,0.5mM TCEP (HEPES buffer) and 1 tablet of EDTA free protease inhibitors (Roche, 11697498001). The cell lysate was centrifuged at 21,000 g for an hour 4°C. The clear supernatant was added to equilibrated GST beads in HEPES buffer for affinity chromatography. The proteins were eluted using 50mM reduced glutathione from the GST beads. The GST-tag was cleaved with PreScission protease at 4°C overnight. The quality of the protein complexes was analyzed using SDS-PAGE and the protein complexes were further purified by gel filtration using Superdex 200 increase 10/300 GL (GE healthcare) and Superose 6 10/300 (GE healthcare) column equilibrated in the HEPES buffer.

For the DII domain deletion experiments, genes encoding 3xmyc-RUVBL1 and 3xmyc-RUVBL1ΔDII were cloned into pRSETA using the NheI sites. The 6xHis-RUVBL2 and 6xHis-RUVBL2ΔDII genes were cloned into pET28b plasmids using the NdeI sites. 6xHis-DII domains of the RUVBL1 and RUVBL2 were cloned into pCDFduet-1 plasmids using NdeI and KpnI sites and the human GST-TELO2 gene was cloned into a pFBDM plasmid using BamHI and EcoRI sites. RUVBL1/2, their ΔDII domain versions, and isolated DII domains were expressed in BL21 (DE3) cells and purified using Talon affinity chromatography in 50mM Tris-HCl, pH 7.8, 300mM NaCl buffer. The proteins were further purified by gel-filtration (using HiPrep Sephacryl S-400 HR and S200 16/60 columns) in the same buffer. GST-TELO2 protein was expressed in Sf9 cells and was purified using GST affinity chromatography in 20mM HEPES, pH 7.85, 500mM NaCl buffer and further purified by gel filtration using Superdex 200 Increase 10/300 GL column.

#### Purification of the mTOR-KD-Lst8 complex and mTOR1-929 (heat repeat)

Human mTOR^ΔN^ (residues 1376 to 2549 with a His-Flag-PreScission cleavable tag at the N terminus) was co-expressed with full length human Lst8 in *Spodoptera frugiperda* Sf9 cells. Cells were harvested and resuspended in lysis buffer containing 20 mM HEPES, pH 7.85, 250 mM NaCl, 1 mM EDTA, 0.5 mM TCEP and supplemented with 1 tablet of EDTA free protease inhibitors (Roche, 11697498001).Cells were lysed, centrifuged at 21,000 g for 60 min at 4°C and then applied to 1 mL of anti-FLAG M2 Affinity Gel-resin (Sigma-Aldrich Ltd) and incubated for 60 min at 4°C on a 200 rpm/min roller. The resin was then washed with 50 mL lysis buffer. The bound mTOR^ΔN^ was released from the resin using 0.2 mg/ml flag peptide (Peptide synthetics) dissolved in lysis buffer.The flag-tag was removed from mTOR-KD by incubating with PreScission protease at 4°C overnight. The mTOR^ΔN^ was then concentrated and further purified by gel-filtration using Superdex 200 increase 10/300 GL column in lysis buffer.

#### Interaction studies using pull-down experiments

For GST-tag affinity, strep-tag and flag-tag pull-down experiments, 40 μL beads were equilibrated in 20mM HEPES, pH 7.85, 140mM NaCl, 0.5mM TCEP, 0.03% CHAPS detergent. 20 μL of 1mg/ml of GST-TTI2-TTI1, yeast and human TTT proteins, 1mg/ml C-terminal Strep-tagged RUVBL2 and 2xFlag- mTOR^N^_,_ KmTOR-KmLst8 were used for pulldown experiments. The subcomplexes were incubated with either 40ul GST/Strep/Flag beads and incubated for 45mins at 4°C rotating at 20 rpm The beads were washed three times with 200 μL buffer by pelleting with quick centrifuge at 3000 g for 10sec. The quality of the pull-down interactions was visualized using 4%–12% SDS-PAGE in MES and MOPS buffer.

For the DII domain deletion experiments, GST beads were equilibrated in 20mM HEPES, pH 7.85, 140mM NaCl and 0.001% Tween 20 buffer. 5 μM GST-TELO2 protein was mixed with 50ul of GST beads. For the pull-down experiments, 2 μM of RUVBL1/2, RUVBL1/2ΔDII and isolated DIIs of RUVBL1/2 were added to the GST-TEL2 sample. For the negative control, 2 μM GST-tag alone was incubated with above proteins. The complex was incubated for 45 mins at 4°C rotating at 20 rpm/min. The beads were washed three times with 500 μL of 20mM HEPES, pH 7.85, 140mM NaCl and 0.001% Tween 20 buffer. The bound protein complexes were boiled in Laemmli buffer and visualized on 4%–12% SDS-PAGE.

#### ATPase assay

The ATPase assay of RUVBL1-RUVBL2 was carried using PiColorLock detection reagent kit (Innova Biosciences Ltd). The ATPase assay was carried out in 50mM Tris-HCl Ph 7.5, 50mM NaCl, 20mM MgCl_2_ 0.1%Glycerol, 0.01% Tween X-100. All the ATPase reactions were carried out in triplicates. For each ATPase reaction, 0.25 μM RUVBL1-RUVBL2, and 1.2 μM-400 μM ATP was used. The rest of the protocol was followed according to manufacturer’s instructions (Innova Biosciences Ltd). For the R2-TTT ATPase assay, 0.25 μM RUVBL1-RUVBL2 and 0.75 μM TTT complexes were mixed with above mentioned buffer and the PiColorLock reagents. The reaction mixture was incubated at 37°C for 30mins. For the negative control, 400 μM ATP alone and/or proteins alone were used. The reactions were scanned at 650nm using CLARIOstar (Biomedical Solutions Inc.) .

#### TOR kinase assay

To measure the effect of TTT and R2TP in mTOR kinase activity, we used 1 μM KmTOR-Lst8 1 μM, 3 μM Rvb1p/2p (hexamer) and 5.5 μM ScTel2-TTi1-TTi2, and 16 μM purified 4EBP1 protein. The kinase assay was performed using HEPES buffer (25mM HEPES, pH 7.5, 50mM KCl, 5mM MnCl2, 5mM MgCl2 in a final volume of 20 μl. The kinase reaction was conducted in the presence of 100 μM ATP at 23°C for 30mins. For the negative control experiment, we used 10 μM ATP competitive mTOR kinase inhibitor Dactolisib (company). The reaction was stopped by the addition of 20mM EDTA followed by the addition of 5 μL Laemmli buffer followed by immediate boiling for 5mins at 100°C. The samples were subjected to standard western blot using 4%–12% SDS-PAGE. The phosphorylated 4EBP1 was detected by using anti-Phospho-4EBP1 (Thr37/46) polyclonal antibody (Cell Signaling Technology, cat. number 236B4). Chemiluminescence reaction was visualized using ECL solution (GE Healthcare) according to the manufacturer’s instructions.

#### Reconstitution of R2TP-TTT and R2-TTT complexes

1 μM of the human and yeast R2TP/RUVBL1-RUVBL2 complexes was assembled according to previously described method (Martino et al., 2018; Rivera-Calzada et al., 2017). 2 μM of pure yeast and human TTT complex were mixed with R2TP/RUVBL1-RUVBL2 complexes and these assembled complexes were incubated for 1 hour at 4°C. The quality of the samples was analyzed using 4%–12% SDS-PAGE and ultimately used for the cryo-grids preparation.

#### Cryo-EM sample preparation and data collection

Freshly assembled preparations of the different complexes (1 μM) were used to prepare cryo-grids by applying 3.7 μL of complex to a holey-carbon grids (Quantifoil™ 1.2/1.3, 300 mesh copper) which was glow discharged in air with PELCO easiGlow by applying a current of 10mA for 60 s. Grids were blotted for 3 s at 25°C and plunged into liquid ethane using FEI-Vitrobot Mark IV (Thermo Fisher Scientific) or Leica EM GP2 (Leica microsystems) and stored in liquid nitrogen prior to imaging. Data were collected using FEI Titan Krios electron microscopes at 300kV, equipped with a Gatan BioQuantum (slit width 20eV) K2 direct electron detectors operating in counting mode. The R2-TTT and R2-T2 (TTi1-TTi2) data were collected at Astbury Biostructure Laboratory, Leeds, UK and R2TP-TTT data was acquired at eBIC Diamond Light Source, UK. Automated data acquisition software EPU (Thermo Fisher Scientific) was used to collect data based on a published protocol (*29*) and with the parameters described in Table S1. Micrograph movies were collected with 40 fractions across a 10 s exposure. Autofocusing was performed after every 10 μm. For R2-T2 and R2-TTT data was collected using total doses of 49.6 e^-^/Å^2^ and 51.2 e^-^/Å^2^ respectively and the R2TP-TTT data was acquired with dose of 42.12 e/Å ^2^ over 40 fractions.

#### Cryo-EM data processing

All datasets (Table S1) were processed following similar strategies. Movies obtained for the different samples and imaging sessions were aligned and local motions were corrected using MotionCor2 (Zheng et al., 2017). The contrast transfer function (CTF) estimation was performed using Gctf (Zhang, 2016). Particle selection, 2D and 3D classifications, refinement and all other image processing steps were performed using the tools provided in Relion 2 (Kimanius et al., 2016) and Relion 3 (Zivanov et al., 2018), unless other program is specified. Particles were automatically picked from the micrographs and bad particles from the initial datasets were removed after several rounds of 2D classification and averaging. Cleaned datasets were then classified in several sub-groups in 3D using initial templates generated using the *ab initio* reconstruction tools in cryoSPARC (Punjani et al., 2017) and the *ab-initio* routine available in Relion. The best sub-groups in each 3D classification were selected for refinement either separately or after merging those classes with similar features. The final structure was then selected and the particles were polished in Relion3.1 before a final round of refinement using shiny particles. All final maps were post-processed and sharpened in Relion and the resolutions estimated in Relion using gold standard Fourier Shell Correlation (FSC) and a cut-off of 0.5 and 0.143 as implemented in Relion. Local resolution ranges for each map were estimated using Relion. Details about the initial and final number of images, estimated average resolution and range of resolutions for each of the maps described in this work are indicated in Table S1.

To determine the structure of the R2-TTT complex, the sample was prepared by saturating R2 with an excess of TTT. We collected two independent datasets in two sessions using the same microscope and imaging conditions which were initially processed separately as detailed in Figure S1. Each dataset was cleaned by successive rounds of 2D and 3D classification to remove bad particles. Remaining particles were then subjected to a round of 3D classification. Some of the 3D classes obtained were directly removed after inspection of the volumes which were of poor quality, while the remaining sub-groups were subjected to image refinement and we selected those classes that refined at higher resolution and where RUVBL1-RUVBL2 was occupied by 3 copies of TTT. In the selected classes, several TTT complexes interacted with each RUVBL1-RUVBL2 hexamer and we implemented a symmetry expansion strategy to make use of all the available information as described before (Martino et al., 2018; Zhou et al., 2015). Briefly, because each R2-TTT complex has a roughly threefold symmetry and contained one, two or three TTT complexes per R2, we rotated each particle around the 3-fold symmetry axis three times to place all TTT complexes in the same position. This operation triplicated the dataset and then we placed a mask around one of the TTT positions and including part of the R2 ring. After symmetry expansion, particles were classified and refined locally using a mask embracing the RUVBL1-RUVBL2 hexameric AAA+ core, the two DII domains interacting with TTT and one copy of TTT. This allowed the reconstruction of the best classes, one for each dataset, at 4.3 and 3.4 Å resolution for dataset 1 and 2 respectively (Figure S2).

In order to improve the resolution of TTT, we merged the particles that were part of the best volumes obtained from each dataset to increase the number of good particles (Figure S1). In these maps, the resolution of the TTT complex was lower than the RUVBL1-RUVBL2 hexamer and we therefore implemented a multi-body refinement strategy (Nakane et al., 2018). We defined two bodies, corresponding to the TTT complex and the two DII domains in contact with TTT (body 1) and the RUVBL1-RUVBL2 hexamer except the two DII domains in contact with TTT (body 2). Multi-body refinement revealed some flexibility in the interaction between TTT and RUVBL1-RUVBL2 and significantly improved the resolution of the density corresponding to TTT. Particles were subjected first to a local classification strategy using a mask including only the density for TTT to select those particles of the best quality and more homogeneous TTT complexes, which were then refined using the multi-body strategy. The resolution of the TTT body was estimated as 5.0 Å using an FSC cut-off of 0.143 (Figure S2). We explored if dividing the TTT body in several smaller sub-bodies could improve resolution, but this attempt proved for most part unsuccessful, with the exception of the region assigned to TELO N-terminal domain. A final composite map was built using the best RUVBL1-RUVBL2 hexameric core corresponding to the single-body refinement of R2-TTT from dataset 2, the best TTT obtained from the merged particles, and the body corresponding to TELO2 N-terminal domain.

To prepare the human R2TP-TTT for cryo-EM analysis, we mixed RUVBL1-RUVBL2 with an excess of RPAP3-PIH1D1 complex using conditions that have been shown before to saturate RUVBL1-RUVBL2 and generate a sample where most R2 is forming R2TP complexes (Martino et al., 2018; Maurizy et al., 2018; Munoz-Hernandez et al., 2019). Images were collected and particles processed using similar methodologies to those described above (Figure S6). Although we could potentially expect detecting images of R2, R2TP, R2-TTT and R2TP-TTT in the micrographs, because we used an excess of RPAP3-PIH1D1 and TTT with respect to RUVBL1-RUVBL2 in the reaction, the mix should be substantially enriched in R2TP-TTT. Accordingly, 2D averages obtained from the images in this experiment only found R2TP-TTT. R2TP could be readily distinguished from RUVBL1-RUVBL2 in the 2D averages by the presence of the C-terminal domain of RPAP3 interacting at the ATPase-face of the RUVBL1-RUVBL2 ring, and TTT was visible as a density attached at the DII face of the ring (Figure S6). 3D classifications also confirmed that all the classes containing good particles corresponded to R2TP-TTT.

#### Model generation, fitting, and visualization

Atomic models for human RUVBL1 and RUVBL2 and RPAP3-RBD, were derived from previous cryoEM and NMR structures (Martino et al., 2018; Maurizy et al., 2018; Munoz-Hernandez et al., 2019) and initially docked as rigid bodies into cryoEM density in Chimera (Goddard et al., 2007). Homology models of the DII domains, which were not fully resolved in previous structures were constructed using I-TASSER (Roy et al., 2010), merged into the experimentally determined RUVBL1 and RUVBL2 models, docked into density using Chimera, and geometrically regularised in Coot (Casanal et al., 2020). A homology model for human TELO2 N-domain was constructed using the I-TASSER server. Side-chains were stripped back to Cβ except for proline residues, and the model docked as a rigid body into density using Chimera. The position and orientation of individual helices, and their connecting loops was manually optimized to the experimental density in Coot. Models for TTI1 and TTI2 were constructed by manual building of poly-alanine α helices into experimental density, using the right-handed solenoid topology of HEAT repeat to determine connections. The polarity of the two chains was subsequently determined by biochemical analyses and the models adjusted accordingly. Based on the length of these proteins, the current TTI1 and TTI2 HEAT-repeat models account for ∼60% and 90% of the amino acid sequences of these proteins, respectively. The fit of the overall model was optimized by real-space refinement in Phenix (Afonine et al., 2018) to a combined map generated from a consensus of the highest resolution overall map, and individual local-resolution filtered multibody-refined volumes (see above). All structural depictions were generated using Chimera.

#### Cross-linking mass spectrometry (XL-MS) of the human R2TP-TTT complex

The 1uM assembled complex of R2TP-TTT (in 20 mM HEPES pH 8.0, 140mM NaCl, 0.5mM TCEP) was cross-linked with a 100-fold excess of isotopically labeled N-hydroxysuccinimide (NHS) ester disuccinimidyl suberate (DSS-H_12_/D_12_, Creative Molecules Inc., Canada), with respect to the protein concentration. The cross-linking reactions were incubated for 60 minutes at room temperature and then quenched by the addition of NH_4_HCO_3_ to a final concentration of 20 mM and incubated for further 15 min.

The cross-linked proteins were reduced with 10 mM DTT and alkylated with 50 mM iodoacetamide. Following alkylation, the proteins were digested with trypsin (Promega, UK) at an enzyme-to-substrate ratio of 1:50 in an overnight incubation at 37°C followed by the addition of Glu-C protease (Promega, UK) at a ratio of 1:10 and incubated for 4 hours at 37°C. After digestion, the samples were acidified with formic acid to a final concentration of 2% v/v and the peptides fractionated by peptide size exclusion chromatography, using a Superdex Peptide 3.2/300 (GE Healthcare) with 30% v/v Acetonitrile/0.1% v/v TFA as mobile phase and at a flow rate of 50 μl/min. Fractions were collected every 2 min over the elution volume 1.0 mL to 1.7 ml. Prior to LC-MS/MS analysis fractions were freeze-dried.

Lyophilized peptides for LC-MS/MS were resuspended in 0.1% (v/v) formic acid and 2% (v/v) acetonitrile and analyzed by nano-scale capillary LC-MS/MS using an Ultimate U3000 HPLC (ThermoScientific Dionex, USA) to deliver a flow of approximately 300 nl/min. A C18 Acclaim PepMap100 5 μm, 100 μm × 20 mm nanoViper (ThermoScientific Dionex, USA), trapped the peptides before separation on a C18 Acclaim PepMap100 3 μm, 75 μm × 250 mm nanoViper (ThermoScientific Dionex, USA). Peptides were eluted with a gradient of acetonitrile. The analytical column outlet was directly interfaced via a nano-flow electrospray ionisation source, with a hybrid dual pressure linear ion trap mass spectrometer (Orbitrap Velos, ThermoScientific, San Jose, USA). Data dependent analysis was carried out, using a resolution of 30,000 for the full MS spectrum, followed by ten MS/MS spectra in the linear ion trap. MS spectra were collected over a m/z range of 300–2000. MS/MS scans were collected using threshold energy of 35 for collision-induced dissociation.

For data analysis, Xcalibur raw files were converted into the MGF format through MSConvert (Proteowizard; (Kessner et al., 2008)) and used directly as input files for StavroX (Götze et al., 2015). Searches were performed against an *ad hoc* protein database containing the sequences of the complex and a set of randomized decoy sequences generated by the software. The following parameters were set for the searches: maximum number of missed cleavages 3; targeted residues K, S, Y and T; minimum peptide length 5 amino acids; variable modifications: carbamidomethyl-Cys (mass shift 57.02146 Da), Met-oxidation (mass shift 15.99491 Da); DSS cross-links mass shift 138.06808 Da (precision: 10 ppm MS1 and 0.8 Da MS2); False Discovery Rate cut-off: 5%. Finally, each fragmentation spectrum was manually inspected and validated.

### Quantitation and statistical analysis

Data were fitted to the Michaelis–Menten equation using non-linear regression in GraphPad Prism Software 8 to calculate the reported Vmax and Km values.

## Supplemental information

Document S1. Table S1 and Figures S1–S6

Document S2. Article plus supplemental information
