# Supplementary material for: Structure of the TELO2-TTI1-TTI2 complex and its function in TOR recruitment to the R2TP chaperone
Source: Cell Rep. 2021 Jul 6;36(1):109317. doi: 10.1016/j.celrep.2021.109317 (PMC8278493; doi:10.1016/j.celrep.2021.109317)
Supplement: Document S1. Table S1 and Figures S1–S6 [file mmc1.pdf]

Cell Reports, Volume *36*

Supplemental information

Structure of the TELO2-TTI1-TTI2 complex and its function in TOR recruitment to the R2TP chaperone

Mohinder Pal, Hugo Muñoz-Hernandez, Dennis Bjorklund, Lihong Zhou, Gianluca Degliesposti, J. Mark Skehel, Emma L. Hesketh, Rebecca F. Thompson, Laurence H. Pearl, Oscar Llorca, and Chrisostomos Prodromou

	R2TP-TTT	R2-TTT Data set 1	R2-TTT Data set 2	TTT from R2-TTT	R2-TT	yR2TP-TTT
**Data collection and processing**						
Magnification	130,000	130,000	130,000	130,000	130,000	130,000
Voltage (kV)	300	300	300	300	300	300
Electron exposure (e–/Å^2)	50	51.2	51.2	51.2	49.6	50.1
Defocus range (μm)	1.20-2.70	1.25-2.5	1.25-2.5	1.25-2.5	1.25-2.50	0.75-2.0
Pixel size (Å)	1.048	1.07	1.07	1.07	1.07	1.07
Symmetry imposed	C1	C1	C1	C1	C1	C1
Initial particle images (no.)	168882	468139	429303	468139	57102	231596
Final particle images (no.)	90575	142043	267149	88340	23518	87438
Map resolution (Å) FSC (0.143)	6.1	4.3	3.4	5.0	9.0	Class1 5.9<br>Class2 10.0
Map resolution (Å) FSC (0.5)	8.5	5.8	4.1	7.8	17.6	Class1 7.3<br>Class2 14.3
Map resolution range (Å)	4.39 - 12	3.72-7.99	3.19 – 8.19	4.07 – 9.9	6.8 – 24.3	Class 1 4.7 – 21.5<br>Class 2 8.7 – 24.6
**Refinement**		using Phenix combine-focused map (EMDB 12979)(PDB 7ole)				
Initial model used (PDB code)		6FO1				
Model res. (Å) FSC (0.143, 0.5)		3.3, 3.7				
Map sharpening *B* factor (Å^2)		110				
Model composition<br>Non-H atoms<br>Protein resi.<br>Ligands		 27830<br>4120<br>6				
B factors (Å^2)<br>Protein<br>Ligand		 160.3<br>42.4				
R.m.s. deviations<br>Bond lengths (Å)<br>Bond angles (°)		 0.007<br>0.899				
Validation<br>MolProbity score<br>Clashscore<br>Poor rotamers (%)		 3.1<br>78.2<br>0.23				
Ramachandran plot<br>Favored (%)<br>Allowed (%)<br>Disallowed (%)		 79.91<br>19.77<br>0.32				

Table S1 – related to Fig. 1 - Cryo-EM data collection, refinement and validation statistics

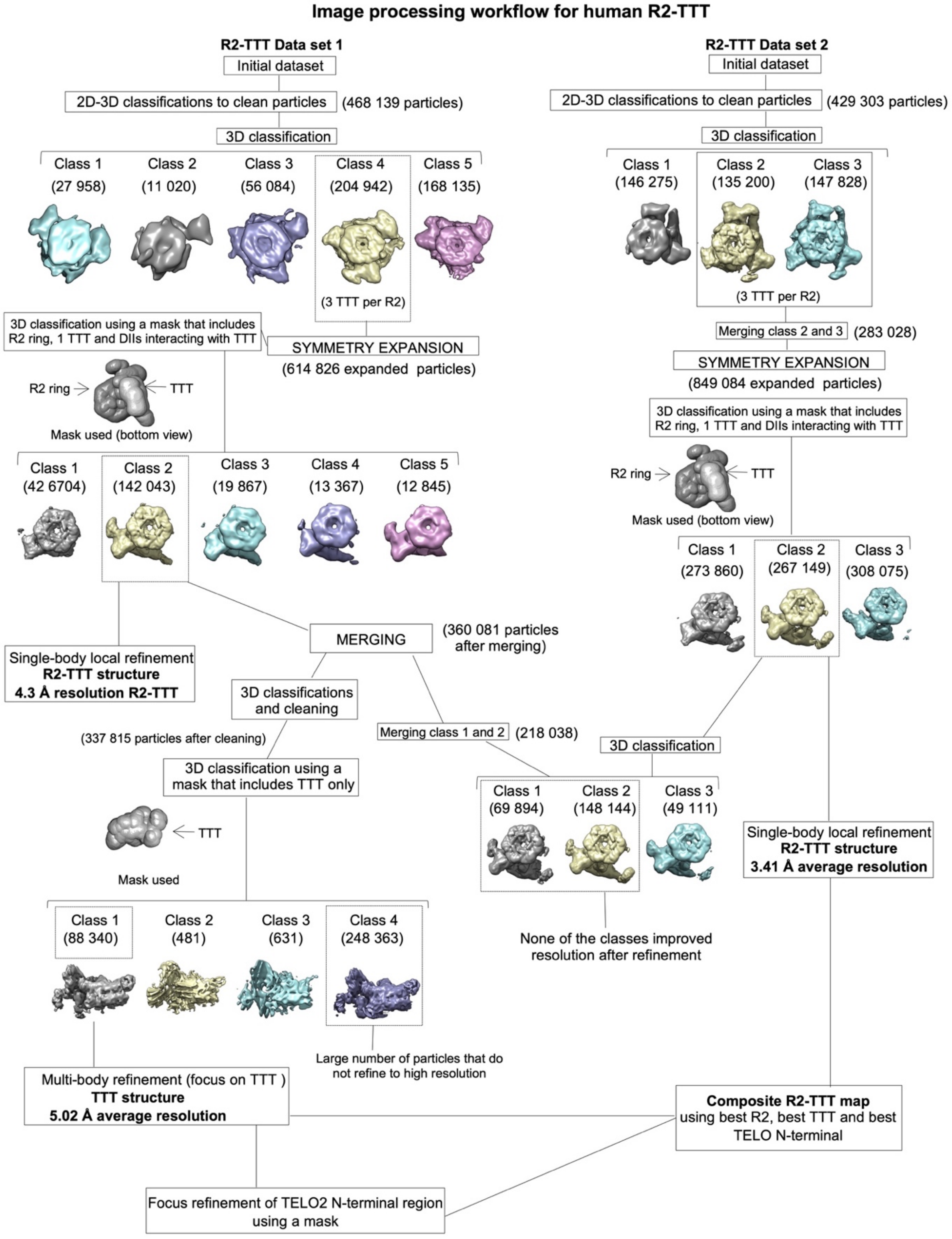

Supplemental Figure 1 – related to Fig 1Human R2-TTT cryo-EM Data Processing workflow

Workflow of the image processing strategy followed to determine the structure of the R2-TTT complex and TTT. Two datasets were obtained in the same microscope, which were processed separately and using a symmetry expansion strategy. The best particles of each group were merged to obtain the best cryo-EM of the TTT region.

Resolution estimation for the cryo-EM of the human R2-TTT complex

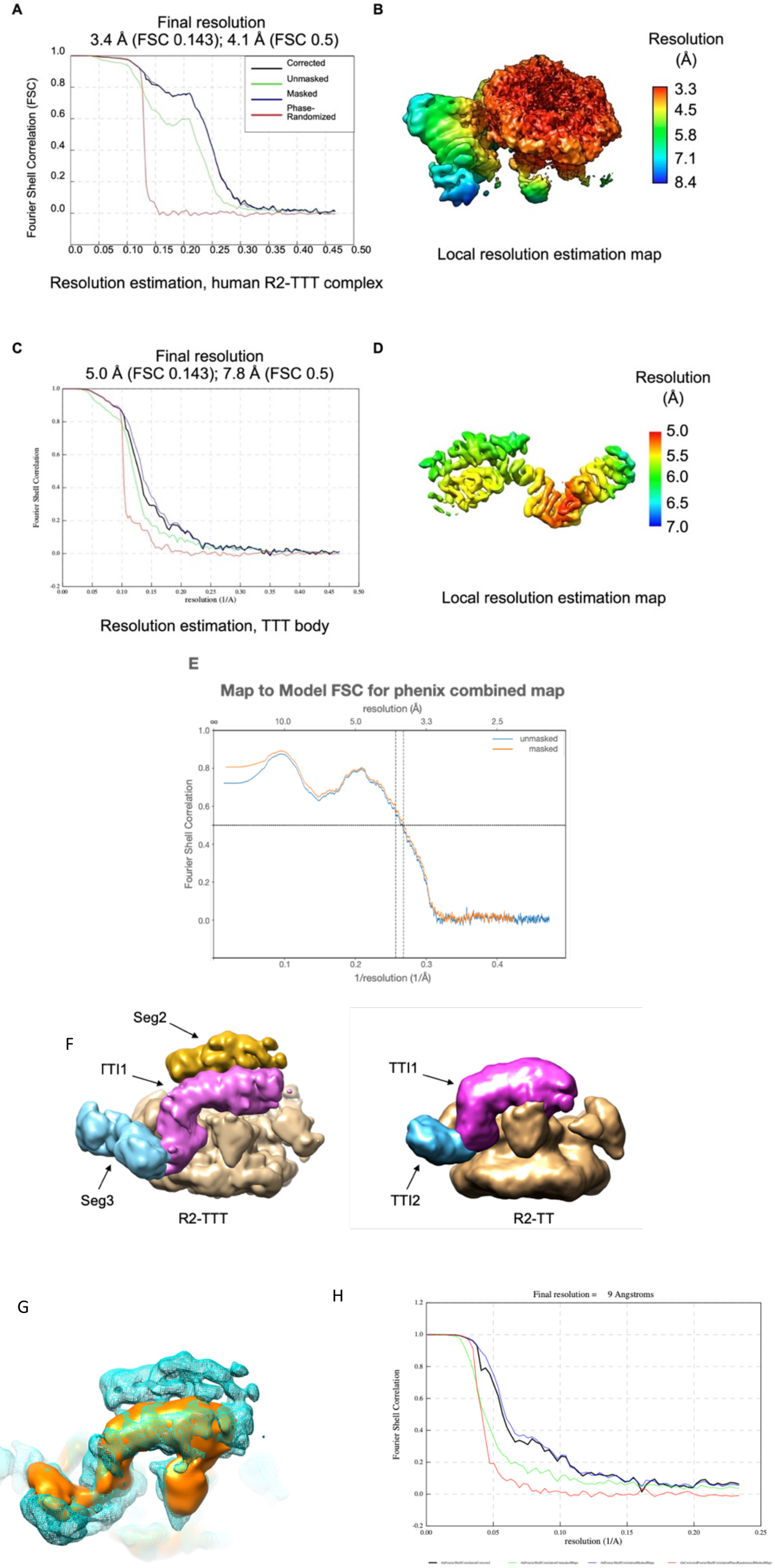

Supplemental Figure 2 – related to Fig 1

(A) Fourier Shell Correlation (FSC) curves estimating the average resolution of the cryo-EM map obtained for R2TP-TTT from data set 2.
(**B**) Local resolution map of R2-TTT. Scale shown in the panel at the right.
(**C**) Fourier Shell Correlation (FSC) curves estimating the average resolution of the cryo-EM map obtained for the TTT body within human R2-TTT after merging particles from data sets 1 and 2.
(**D**) Density for the TTT segment after MultiBody refinement. The density shows three clear segments of tubular density corresponding to □-helices arranged in a right-handed solenoid super-secondary structure. The surface is coloured according to the estimated resolution and scale shown in the right-hand panel.
(**E**) Map-to-model FSC curve of R2-TTT calculated in phenix for the combined map
(**F**) Comparison of cryo-EM structure of (left) R2-TTT complex (5.4Å resolution) with (right) R2-TT complex (~9Å resolution), showing the absence of Seg2 (gold) in the R2-TT complex from which TELO2 was omitted. Both maps are shown filtered to 9Å to facilitate comparison.
(**G**) Close-up of the TT(T) region of the superimposed R2-TTT and R2-TT maps – R2-TTT (cyan), R2-TT (gold).
(**H**) FSC curve for R2-TT map.

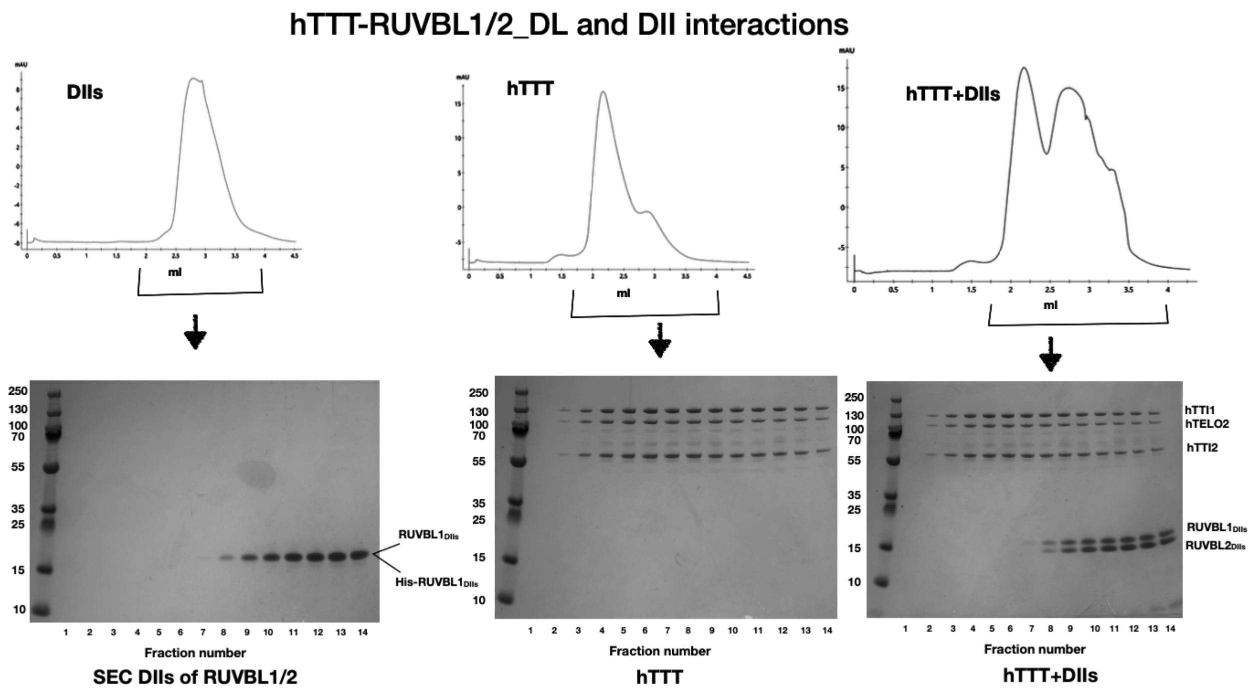

Supplemental Figure 3 – related to Fig 2

Size exclusion chromatography of human TTT and isolated human RUVBL1 and RUVBL2 DII domains. The gel filtration profile of Superose6 5/150 column calibrated in 20mM HEPES, 200mM NaCl, pH 7.85 shows that the DII domains (5uM) elute around 3ml volume in the fraction numbers 7-14. The TTT complex (5uM) alone run on the SEC around 2.3ml as a single peak (in fraction 2-13). When the TTT and DIIs of RUVBL1 and RUVBL2 mixed and applied on the gel filtration column, they eluted in two separate peaks. The SDS-PAGE clearly shows that TTT and DII domains elutes in the same fractions as they were eluted in isolation. This experiment suggests that the DII domains needs their intact AAA+ATPase hetero-hexameric ring to facilitate their interactions with TTT complex.

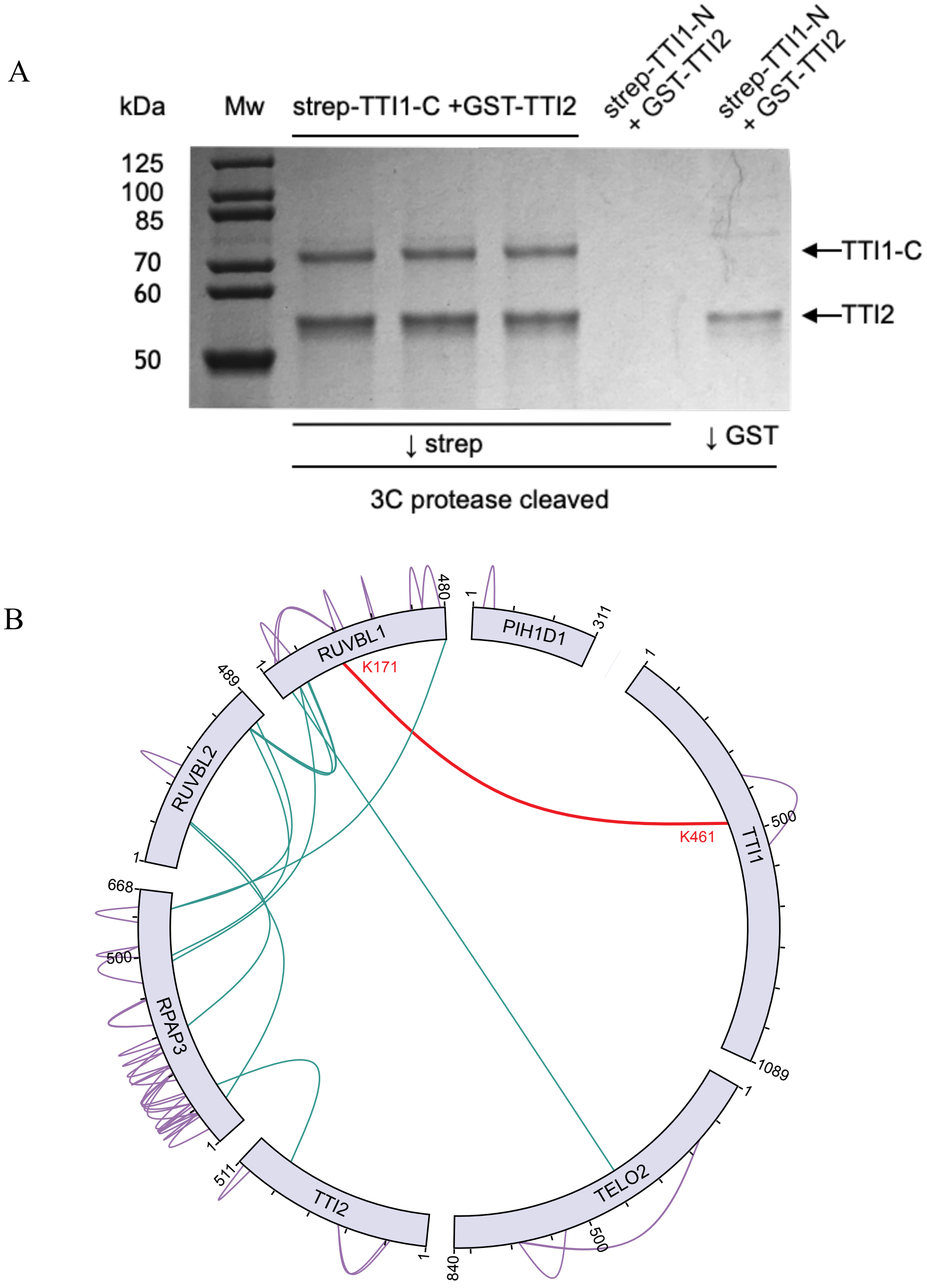

Supplemental Figure 4 – related to Fig 3

A. Coomassie stained SDS-PAGE gel of a pull-down experiment showing co-precipitation of TTI2 (expressed as GST-TTI2) from insect cell lysates when co-expressed with a strep-tagged C-terminal construct of TTI1 (residues 468-1089), but not when co-expressed with a strep-tagged N-terminal construct of TTI1 (residues1-459), which itself expressed little soluble material. GST-TTI2 was nonetheless recovered from cells with the TTI1-N construct, in a GST pull-down. In all cases, strep and GST tags were removed from precipitated material by treatment with 3C protease prior to loading on the gel.

B. Interaction plot from cross-link mass spectrometry analysis of R2TP-TTT complex (see METHODS). Intramolecular links are shown in magneta, and intermolecular contacts in green. The cross-link between Lys 171 or RUVBL1 and Lys 461 of TTI1, that informs the arrangement of these portions deduced from the cryoEM density, is highlighted in red.

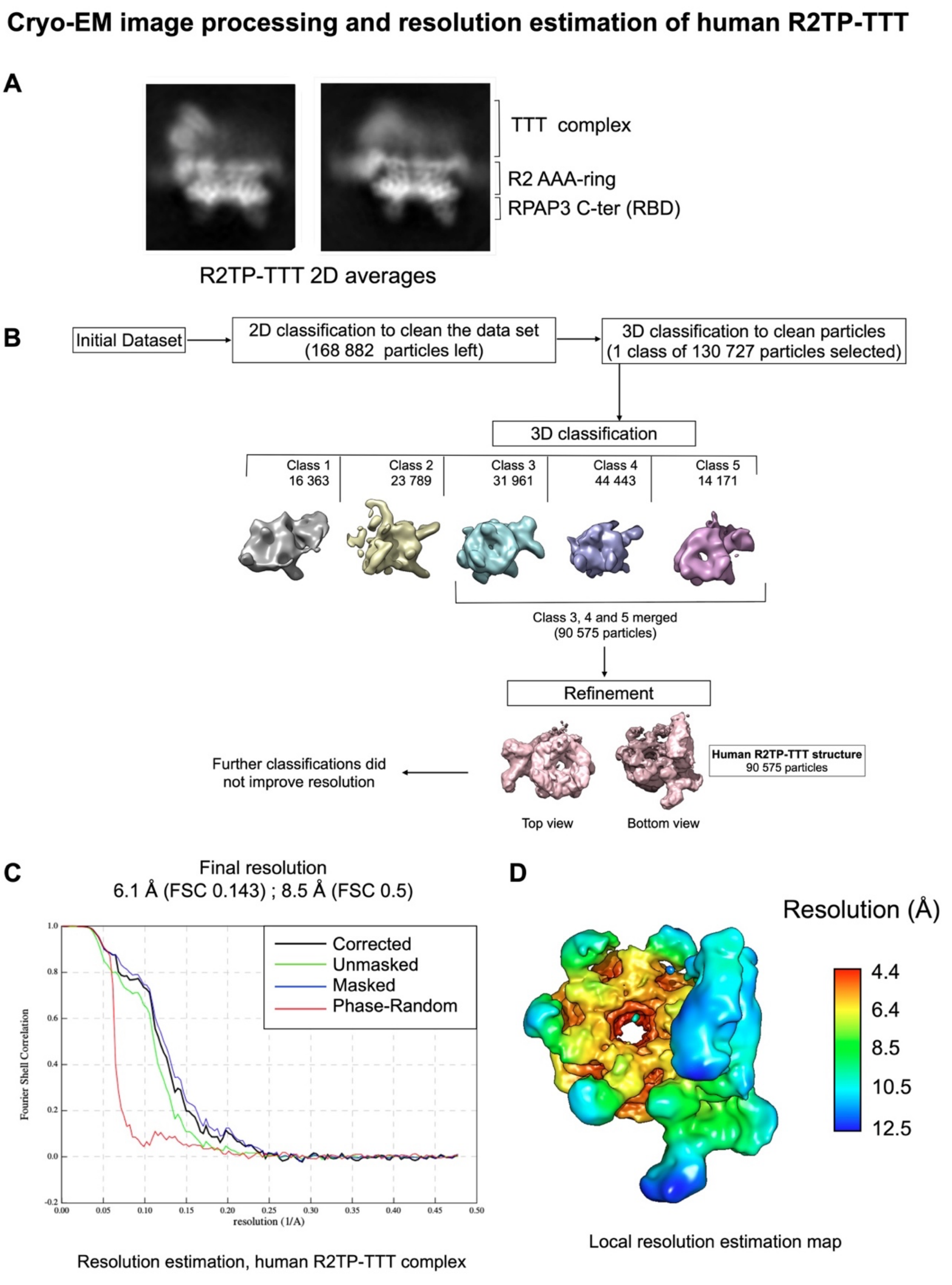

Supplemental Figure 5 – related to Fig 4

Cryo-EM image processing and resolution estimation for human R2TP-TTT

- **A.** Representative 2D averages for R2TP-TTT, indicating the positions of the TTT complex, the AAA-ring of RUVBL1-RUVBL2 (R2) and the RBD domain of RPAP3.
- **B.** Workflow of the image processing strategy followed to determine the structure of the R2TP-TTT complex.
- **C.** Fourier Shell Correlation (FSC) curves estimating the average resolution of the cryo-EM map obtained for R2TP-TTT.
- **D.** Local resolution map of R2TP-TTT. Scale shown in the panel at the right.

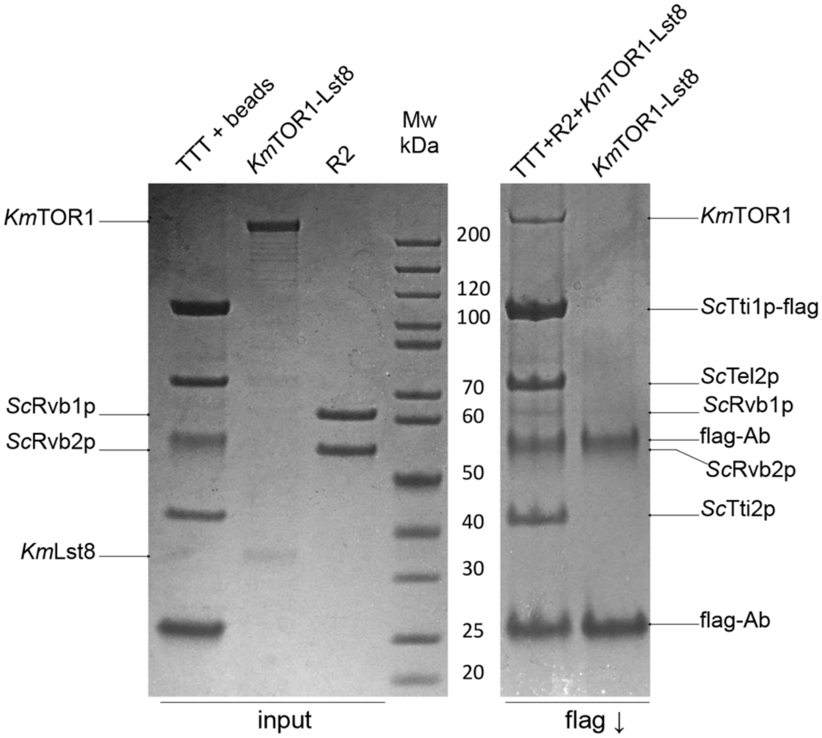

Supplemental Figure 6 – related to Fig 5

Tti1p-Tti2p-Tel2p interacts simultaneously with KmTor and Rvb1p-Rvb2p

Coomassie stained SDS-PAGE gel of a pull-down experiment showing simultaneous co-precipitation of *K.marxianus* TOR and *S.cerevisiae* Rvb1p-Rvb2p by *S.cerevisiae* Tti1p-Tti2p-Tel2p in which the Tti1p carries a flag-tag. KmTOR itself was not precipitated by the flag resin. The lanes are from the same gel which has been split in the image to facilitate labelling of the molecular weight markers
